# You Should Look a Gift Ungulate in the Mouth: Using 2D Occlusal Cheek Tooth Morphology to Study the Evolution of Molarization in Ungulates

**DOI:** 10.1093/iob/obag025

**Published:** 2026-05-30

**Authors:** A J Ashbaugh, H A Jamniczky, J M Theodor

**Affiliations:** Department of Biological Sciences, University of Calgary, Calgary, AB, T2N 1N4, Canada; Department of Cell Biology and Anatomy, University of Calgary, Calgary, AB, T2N 4N1, Canada; Department of Biological Sciences, University of Calgary, Calgary, AB, T2N 1N4, Canada

## Abstract

Cheek teeth are filled to the cusp with information about mammalian evolution. Studying the evolution of mammalian cheek tooth crown complexity has benefited our understanding of mammalian evolution in developmental, morphological, and ecological contexts. Most work is focused on individual cheek tooth loci as opposed to considering the premolars and molars as serial homologues. This focus on individual tooth loci has left the exploration of inter-regional phenomena understudied. One such phenomenon is the molarization of premolars across hoofed mammals; some have simple unicuspid premolars while others have premolar crowns that are equal in complexity to their molars. Many developmental models have been proposed to understand cheek tooth evolution, but minimal work has been done to synthesize these models into a holistic understanding of cheek tooth crown complexity evolution. We investigated if applying a synergized theoretical framework of the inhibitory and patterning cascade model to artiodactyl and perissodactyl taxa could be used to study the evolution of molarization in hoofed mammals. We applied an existing 2D landmarking scheme for the upper and lower premolar molar boundaries of hoofed mammals to capture the morphology across this important identity boundary. Shape data were analyzed through phylogenetically informed modularity analyses to capture the covariation structure at the upper and lower premolar-molar boundaries. A-priori modularity hypotheses were proposed based on developmental models including the patterning cascade model and the inhibitory cascade model. Both artiodactyl and perissodactyl results showed support for modularity across the upper and lower premolar molar boundary but showed more variation in the upper premolar molar boundary. Artiodactyls show consistency in support for modularity hypotheses between upper and lower premolar molar boundaries where perissodactyls show significant differences in support for modularity hypotheses between upper and lower premolar molar boundaries. Our results illustrate that the covariation structure at the premolar molar boundary has convergent and divergent elements that both have consequences for our understanding of the evolution of molarization within and between artiodactyls and perissodactyls.

## Introduction

Precise occlusion between the upper and lower teeth differentiates mammals from other vertebrates. Dentition in other vertebrates functions primarily in food acquisition, but few groups outside of mammals use complex interactions between the upper and lower dentition to initiate mechanical digestion (Ungar 2010; Grossnickle et al. 2022). Mammalian incisors and canines function primarily in food acquisition while the premolars and molars together (collectively known as cheek teeth) have evolved to accommodate specific mammalian dietary niches. Stem and early mammals were heterodont, but they had minute differences in occlusal complexity between the premolars and molars. As mammals evolved, the occlusal crown complexity differences between the premolars and molars became more pronounced (Cifelli and Madsen 1998; Luo et al. 2004; Luo 2007; Davis 2011). The evolutionary potential for differentiation between the premolars and molars has been cited as the crux of mammalian dental evolvability (Stock 2001; Moustakas et al. 2011; Harano and Asahara 2024) and makes mammal dentition an ideal system to study evolution at both the micro and macro scale (Lillegraven et al. 1979; Luo et al. 2001; Moustakas et al. 2011; Wakamatsu et al. 2014).

As environments change, the food resources that are available change. Ungulates illustrate this relationship between their ecology and cheek tooth morphology (Janis and Ehrhardt 1988; Damuth and Janis 2011; Fraser and Theodor 2013; Schulz et al. 2013; Fraser et al. 2015) as they are typically herbivorous and have diets that require variation in foraging behaviours (DeMiguel et al. 2008; Damuth and Janis 2011; Schulz et al. 2013). Browsers eat calorie dense vegetation (e.g., fruits, vegetables, buds, leaves) that is not abrasive and is heterogeneously distributed in the environment. Grazers eat calorie poor vegetation (grasses and sedges) which is abrasive and requires extensive mechanical and chemical digestion (Ungar 2010). Cheek tooth morphology can be used to infer whether a given dentition belonged to a browser, grazer or mixed feeder. For browsers, cheek tooth crown width and sharpness are increased to rupture cell walls (Fortelius and Solounias 2000; Franz-Odendaal and Kaiser 2003). Grazers are subject to increased tooth wear from the abrasive properties of their diet and the environment. Increased tooth wear has resulted in the evolution of increased cheek tooth height (hypsodonty) to counteract the accelerated loss of tooth volume in processing abrasive vegetation (Damuth and Janis 2011; Kaiser et al. 2013). The selective pressures operating on browsers and grazers can target individual teeth for mechanical efficiency, but these same pressures also target cheek teeth collectively as a biomechanically integrated module (Damuth and Janis 2011; Kaiser et al. 2013; Witzel et al. 2018).

**Fig. 1:**
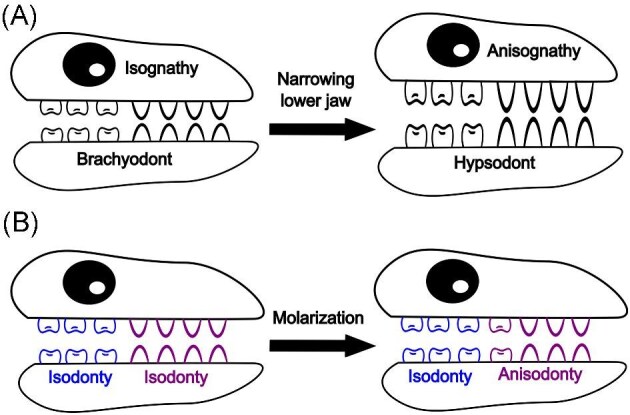
Illustration of the two spectra of variation in studying cheek tooth morphology in ungulates using case study examples, (A) Transition in cheek tooth morphology from brachydont to hypsodont dentition as a product of narrowing the lower jaw and (B) Transition in cheek tooth morphology as a product of premolar molarization. Purple shaded teeth are premolars while blue shaded teeth are molars.

The evolution of ungulate cheek teeth as a biomechanical module has been studied in two ways: (1) study of the differences in morphology between the upper and lower cheek teeth (as a morphological product of isognathy and anisognathy; (Reilly et al. 2001; Kaiser and Fortelius 2003; Avedik et al. 2023) and (2) study of the differences in tooth morphology within a dental row (Fig. 1; isodonty and anisodonty; (Franz-Odendaal and Kaiser 2003; Kaiser and Fortelius 2003; Winkler and Kaiser 2015; Brocklehurst and Benson 2021). Isodonty can be described as a specialized form of homodonty where the morphology of sequential cheek teeth are identical (Fig. 1A), while anisodont cheek teeth show discontinuities between sequential cheek teeth (Fig. 1B). Isodonty and anisodonty have been applied to understand the evolution of overall cheek tooth biomechanics in most cases (Franz-Odendaal and Kaiser 2003; Kaiser and Fortelius 2003; Winkler and Kaiser 2015). However, developmental studies have shown that there are nested levels of regulation of cheek tooth patterning that have the macroevolutionary potential to cascade from the shared origin of the cheek teeth to the development of signalling centers on individual cheek teeth (Jernvall 2000; Kavanagh et al. 2007; Jheon et al. 2013; Ortiz et al. 2018; Yu et al. 2020; Shroff et al. 2024). This cascading effect reveals a gap in the literature—as of the publication of this manuscript, no studies exist that have applied isodonty and anisodonty both within and between dental regions to better understand macroevolutionary changes in cheek tooth morphology. The molarization of premolars, where a premolar shows morphology typical of a molar rather than a premolar, is an ideal example of a shift from an isodont to an anisodont condition within the premolars (Fig. 1; Butler 1952; Holbrook 2015; Selig and Silcox 2022; Ashbaugh et al. 2025).

Molarization can be observed throughout mammals, but most of the literature dedicated to this topic pertains to ungulates (Butler 1952; Holbrook 2015; Bai et al. 2019; Ashbaugh et al. 2025). Early artiodactyls and perissodactyls retain the generalized tribosphenic form in their lower molars with most premolars having few occlusal cusp or cuspid features in comparison to the molars (Rose and Archibald 2004; Prothero and Foss 2007; Rose et al. 2014). Over time, parallel evolution of molarization occurred between artiodactyls and perissodactyls leading to a spectrum of molarization of premolars. The impacts of the evolutionary, developmental, and functional contexts of cheek tooth morphogenesis on the evolution of cheek tooth crown complexity are rarely considered together within the framework of one study, in spite of the ubiquity of the phenomenon (Ashbaugh et al. 2025).

New developmental work has shown great promise in studying the evolution of cusp crown complexity evolution across the cheek teeth. Evidence from noctilionoid bats has been leveraged to argue that mammalian premolars and molars have independent patterning cascades permitting independent modifications to morphogenesis in these dental regions (Sadier and Soukup 2023; Sadier et al. 2023). The foundational work emphasizing the importance of these independent cascades is the inhibitory cascade model (ICM; Kavanagh et al. 2007). The ICM states that there is a cascading effect from the first molar to the second molar to the third molar that impacts the morphogenesis of the molars based on the size of the first molar. However, many mammalian taxa are now known to diverge from this model including many primates, rodents, carnivorans, and ungulates (Polly 2007; Carter and Worthington 2016; Ortiz et al. 2018; Roseman and Delezene 2019; Vitek et al. 2020; Stenberg et al. 2024). A product of these divergences suggests that the ICM is part of a larger patterning system with a stronger role in early molariform morphogenesis, but its relationship to another model, the patterning cascade model (PCM), is not yet clear.

**Fig. 2:**
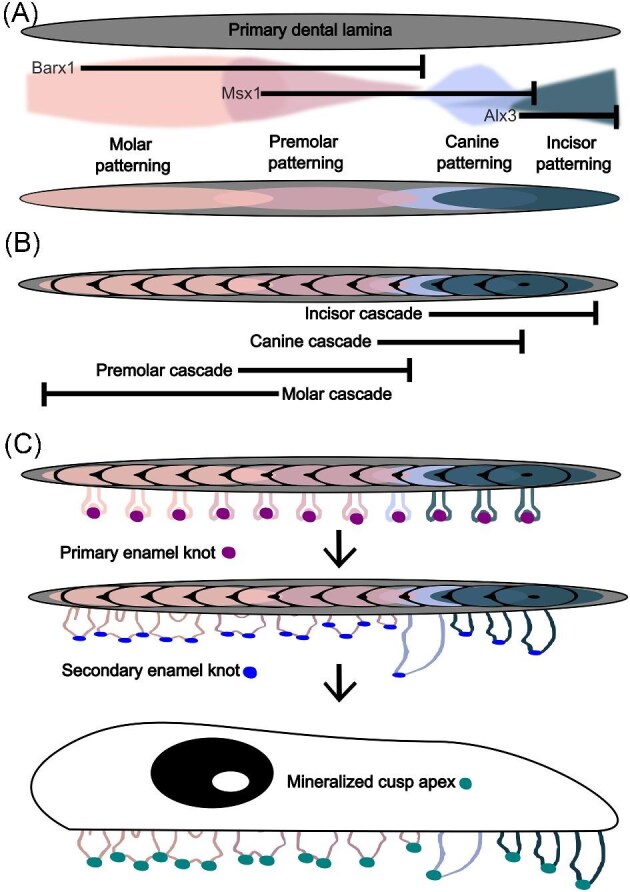
Visualization of the patterning cascade model including (A) Homeobox patterning of the primary dental lamina leading to the regionalization of the dental lamina into incisor, canine, premolar, and molar development zones, (B) Development of individual tooth buds that each have a zone of inhibition that is regulated by the appropriate dental region patterning cascades, and (C) The enamel knot cascades showing how individual tooth loci acquire primary and secondary enamel knots leading to the mineralized cusp apex that will be erupted later in life. Note that (C) is a simplified process of dental development for the purposes of illustrating the potential for cascading information from the dental lamina all the way to the mineralized erupted permanent dentition. For further information on the transmission of patterning information from dental lamina to erupted tooth see (Jheon et al. 2013; Yu et al. 2020; Shroff et al. 2024). Images adapted from Butler 1937, 1939; Osborn 1978; and Wakamatsu et al. 2019.

The PCM proposes that molariform cusp complexity is not predetermined, but is rather a product of interplay between the spatial and temporal development of cusp or cuspid morphogenetic signaling centers prior to mineralization (Fig. 2; Jernvall 2000; Salazar-Ciudad and Jernvall 2002; Ortiz et al. 2018). The important signalling centers in dental development are the primary and secondary enamel knots—clusters of mitotically inert epithelial cells that release morphogens to control the folding of the future occlusal surface (Thesleff and Jernvall 1997; Keränen et al. 1999; Jernvall 2000). Enamel knot signalling is regulated by activator-inhibitor mechanisms which emphasize the importance of spatiotemporal organization of developmental tissues prior to, during, and after dental morphogenesis initiates (Osborn 2008; Jheon et al. 2013; Soldatov et al. 2019; Yu et al. 2020; Guo et al. 2022; Jing et al. 2022; Shroff et al. 2024). The PCM has only recently been extended beyond primates to understand cuspid development as it relates to evolution of lower cheek tooth crown complexity in bears (Stenberg et al. 2024) and ungulates (Ashbaugh et al. 2025). The PCM has yet to be applied to the upper cheek teeth of non-primate mammals to better understand the role of occlusal function and how that influences the spatiotemporal regulation of cheek tooth crown complexity evolution.

**Fig. 3:**
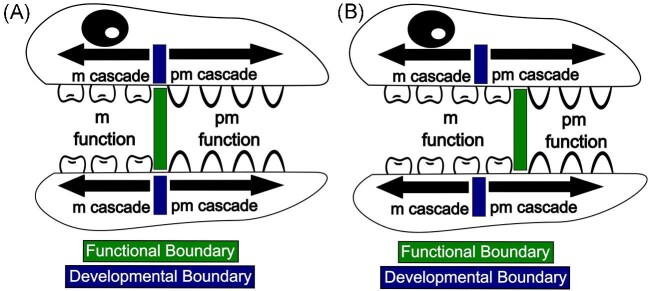
Graphical abstract adapted from Ashbaugh et al. (2025) for the extension of molarlike patterning to premolar loci in the adult dentition of ungulates at the upper and lower premolar molar boundary from (A) non-molarized to (B) molarized morphology. The green boundary represents the functional boundary between the premolars and molars. The blue boundary represents the patterning cascade boundary between the premolars and molars.

The ICM, PCM, and the evolution of molarization should be studied in an integrated framework to better understand the evolution of crown complexity (Fig. 3). We asked the following research question to frame our study: Does applying these developmental models in an integrated morphometric framework to the upper and lower premolar molar boundary result in consistent or diverging covariation structures within and between artiodactyls and perissodactyls? Using a previously validated 2D geometric morphometric (GMM) landmark scheme across the premolar molar boundary, we evaluated the covariation of cusp morphology using various a-priori modularity hypotheses.

## Materials and methods

### Specimen sampling and imaging

Phylogenetic comparative methods were used to approach our research question (see modularity analyses), so the specimen sampling in our study relied on previously published phylogenies of artiodactyls and perissodactyls (Fraser et al. 2015). These phylogenies are composite trees including extant and extinct taxa for both artiodactyls and perissodactyls (Janis et al. 1998; Price et al. 2005; Maguire and Stigall 2009; Fraser et al. 2015). We modified the tip-dates and time-scaling of these trees to incorporate the most recent updates to first and last occurrence data from the Paleobiology Database (https://paleobiodb.org/#/;  Alroy et al. 2001; Uhen et al. 2013). Specimens sampled must have been present on these trees for us to include them in our study. Nexus files for these updated trees can be found in our supplemental materials (Supplementary Materials 1).

Several exclusion and inclusion criteria were applied to sample artiodactyls and perissodactyls. Only one representative specimen per species was selected to ensure that the generated morphospace would not be weighted towards species that are overrepresented in museum collections. Only mature specimens with a fully erupted permanent fourth premolar were included. Specimens with minimal occlusal wear on the premolars and molars were prioritized for inclusion to be able to identify cusps and cuspids for landmarking. In addition, specimens had to have four identifiable cusps on the P4/p4 and M1/m1 to ensure landmark standardization. Lastly, the upper or lower premolar molar boundaries must have been in-situ within the maxilla or mandible respectively to minimize error influencing the position of the premolar molar boundary. Our final sample included 45 upper artiodactyl, 18 lower artiodactyl, 30 upper perissodactyl, and 30 lower perissodactyl premolar molar boundaries. Specimens were mostly sampled and imaged from the American Museum of Natural History Zoology and Paleontology collections with additional specimens sourced from our previous work on this topic (Ashbaugh et al. 2025). All specimens used in our study are documented in the supplement (Supplementary Materials 2).

We took occlusal images of each specimen using an IPhone 14 Pro (48MP Main: 24 mm, ƒ/1.78 aperture). Right mandibles with the premolar molar boundaries in focus were placed on an imaging surface with the occlusal surface perpendicular to the line of sight of the lens to minimize parallax. If a specimen lacked the right premolar molar boundary but had an intact left premolar molar boundary that passed all exclusion and inclusion criteria, images were taken of the left premolar molar boundary and then reflected prior to landmarking.

### 2D GMM, landmarking, and PCA

**Fig. 4:**
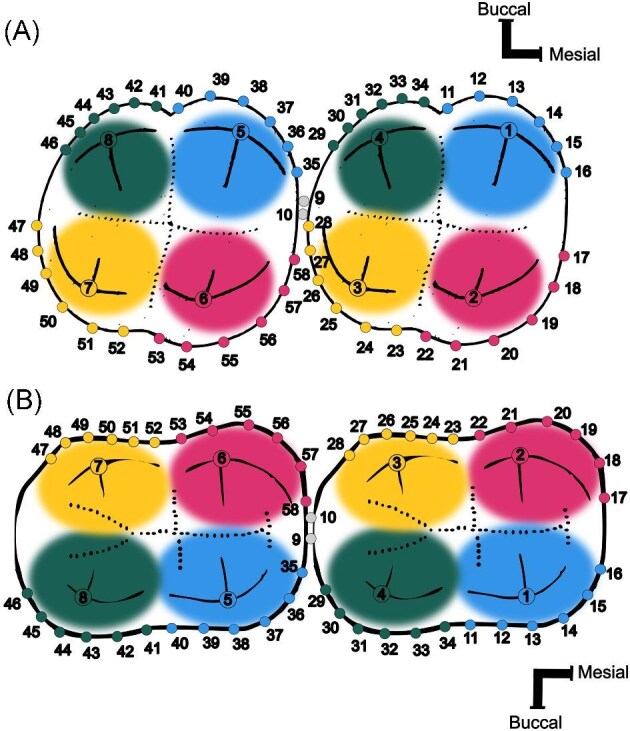
2D geometric morphometric landmark scheme used on the (A) upper and (B) lower premolar molar boundary adapted from Ashbaugh et al. (2025). The total landmark scheme consists of 58 landmarks and are described in the supplemental materials accompanying this manuscript (Supplemental materials 3). Colours are used to demarcate the influence of each enamel knot on cuspid morphology.

The landmark scheme includes 58 landmarks to capture cusp or cuspid covariation across the premolar molar boundary by landmarking the placement of the cusps or cuspids and their respective contours. This landmark scheme has been validated in our previous work (Ashbaugh et al. 2025), but modifications were required to apply the scheme to the upper premolar molar boundary. The landmark scheme was developed for the lower premolar molar boundary with an emphasis on the importance of the trigonid and talonid in the development of the lower cheek teeth (Fig. 4; Ashbaugh et al. 2025). The upper premolars and molars of ungulates have a functional equivalent of the trigonid (trigon) but lack the talonid seen in most ungulate lower molariform cheek teeth. To address this issue, we mirrored the lower landmark scheme to the upper cheek teeth and re-defined landmark placement based on anatomical cusp or cuspid placement to generate the modified upper premolar molar boundary landmark scheme (Fig. 4). This modified landmark scheme retains the same focus as the original—the landmarks are designed to gather information about the functional relationship of the cusps and cuspids across the premolar molar boundary and how they relate to the ICM and PCM. The upper and lower premolar molar boundary landmark scheme with detailed descriptions of landmark placement and visual aids for landmarking can be found in the supplement (Supplementary Materials 3). All specimens were landmarked using the appropriate landmark scheme and then the four shape data sets (upper artiodactyls, lower artiodactyls, upper perissodactyls, lower perissodactyls) were translated, rotated, and scaled to the same size by applying a Procrustes transformation to minimize the impact of size differences on shape differences (Zelditch et al. 2012).

Our sample of species included selenodont, lophodont, and equid or equid-like cusp/cuspid morphology. We ran principal component analyses (PCA) as an exploratory approach to study the principal axes of cusp shape variation across the upper and lower premolar molar boundary in our samples. We found clear separation among the various cheek tooth morphotypes, demonstrating that our landmark scheme could accurately capture the premolar molar boundary for all boundary morphotypes studied (Supplementary Materials 4).

### Modularity analyses

The modularity hypotheses tested across the premolar molar boundaries were similar to those proposed in our previous work to ensure consistency across our studies (Ashbaugh et al. 2025). We will briefly review the tested modularity hypotheses and highlight any modifications required to consider both the upper and lower premolar molar boundary.

**Fig. 5:**
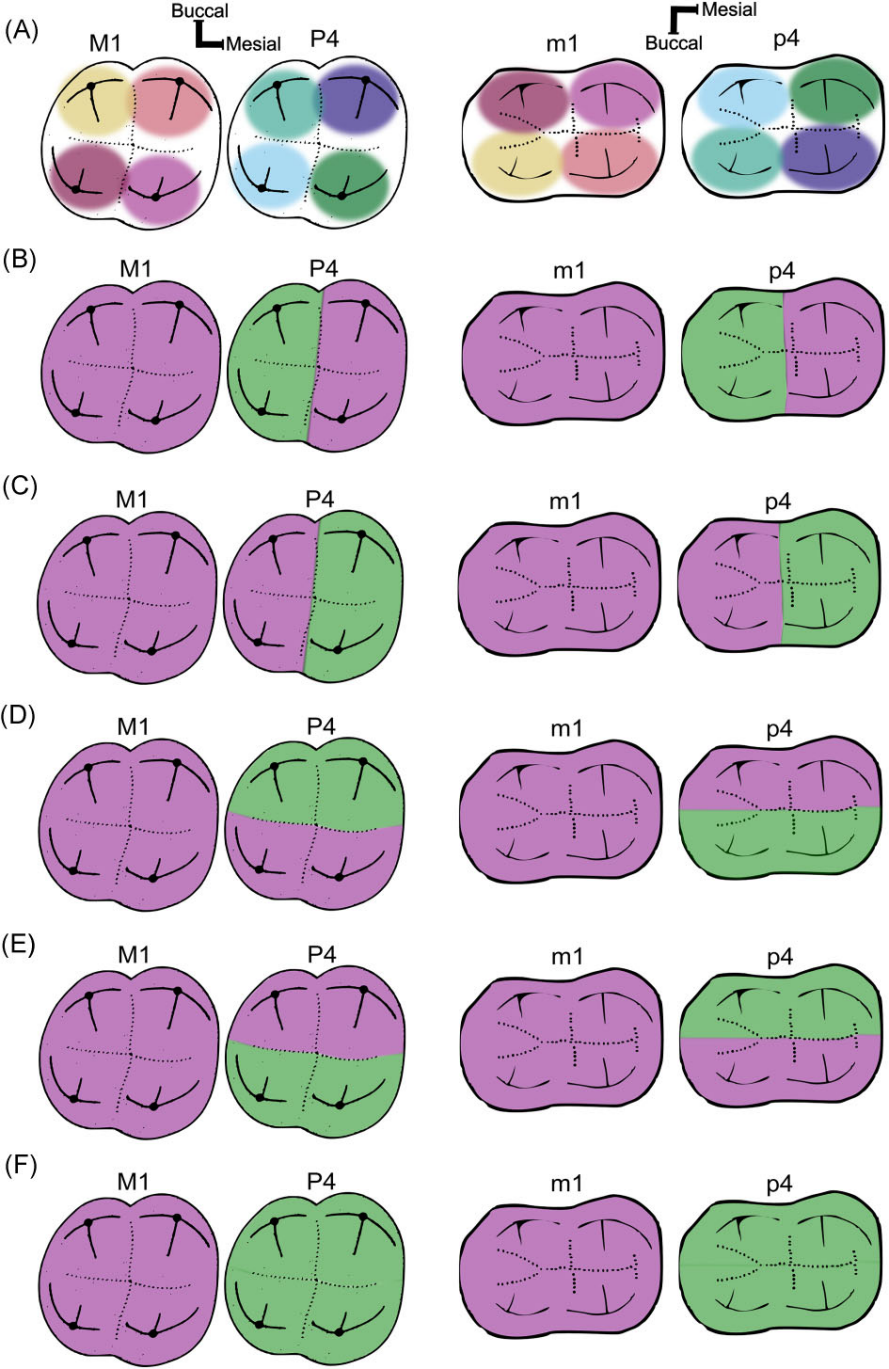
Modularity hypotheses used in this study; (A) Validation hypothesis, which serves as a validation of our landmark scheme; (B) Mesial and (C) Distal hypothesis respectively, which focus on occlusal function between the upper and lower cheek teeth in relation to potential selective pressures within the PCM; (D) Buccal and (E) Lingual hypothesis respectively, which focus on transverse mastication function as it relates potential selective pressures within the PCM; (F) Independence hypothesis, which serves as a proxy for recent work suggesting the premolars and molars have independent patterning cascades. More information on the biological context of these hypotheses is present in the Methods section of this manuscript.

Our first hypothesis (Validation hypothesis) was proposed to ensure the placement of the cusp or cuspid apex is associated with the maximum buccal or lingual contour of each cusp or cuspid—strong association between these landmarks shows evidence of the patterning cascade model operating from cusp apex to base of the cusp (Ashbaugh et al. 2025; Fig. 5A).

The Mesial and Distal hypotheses capture the covariance of the anterior and posterior cusps and cuspids with the first molar morphology (Ashbaugh et al. 2025; Fig. 5B and 5C). Previously, these hypotheses were framed as the Trigonid and Talonid modularity hypotheses respectively to capture the two regions of the tribosphenic molar in occlusion (Ashbaugh et al. 2025). To collect biologically meaningful shape data between the upper and lower premolar molar boundary, these hypotheses required revisions. The occlusal interactions orchestrated by the mesial and distal components of the cheek teeth largely determine whether an ungulate is capable of grazing or browsing, raising the question of how the mesial and distal components covary across the upper and lower premolar molar boundary (Schultz et al. 2021; Avedik et al. 2023). The biological context of these hypotheses remains the same in the present study—the mesial and distal components of the upper and lower cheek teeth have different roles in occlusion which could be the focus of selective pressure in the context of the PCM (DeMers and Hunter 2024; Hunter et al. 2024; Stenberg et al. 2024). The Buccal and Lingual modularity hypotheses are unchanged from our previous work (Fig. 5C and 5D; Ashbaugh et al. 2025). The buccal and lingual cusps and cuspids collectively impact the chewing cycle in mammalian mastication, but are of particular importance in the transverse nature of ungulate mastication (Butler 1939, 1956, 1972; Couzens et al. 2021; Grossnickle et al. 2022). The importance of the buccal and lingual cusps and cuspids as a unit in transverse mastication warrants the study of these cusps and cuspids in the premolars and how they may covary with molar morphology within the context of the PCM.. The Mesial, Distal, Buccal, and Lingual modularity hypothesis are proposed to be nested within the PCM as it relates to covariation across different dental regions (Ashbaugh et al. 2025). Collectively, these hypotheses serve to identify if the PCM has potential as a guiding developmental framework for understanding how inter-regional interactions may influence how molarization evolved in hoofed mammals. We therefore predict that all four of these modularity hypotheses should be significantly supported relative to the null of no modularity across the premolar molar boundary.

The Independence modularity hypothesis is also unchanged from our previous work (Fig. 5E; Ashbaugh et al. 2025). If independent patterning cascades are a contributor to the evolution of crown complexity evolution seen between the premolars and molars in mammals, we predict that both artiodactyls and perissodactyls should show significant support in the performance of the Independence hypothesis. However, we also predict that there should be a relative difference between artiodactyls and perissodactyls in the performance of the Independence modularity hypothesis. Perissodactyl molar ratios fail to be explained by the ICM while most artiodactyls have molar ratios that align with the ICM (Polly 2007; Halliday and Goswami 2013). We predict that the conformity to the ICM should manifest in our modularity results—artiodactyl modularity results should show more support for the Independence hypothesis in comparison to perissodactyls. The Independence hypothesis provides a pathway to evaluating the ICM within the framework of the PCM in two ways not attempted before—(1) between the cheek teeth and (2) between the upper and lower cheek teeth.

All modularity hypotheses were tested for the four shape data sets independently. If a significant phylogenetic signal was found in 2D geometric morphometric data using the composite phylogenies mentioned previously (evaluated using K_mult_; Adams 2014), modularity analyses were conducted with phylogenetic comparative methods (Klingenberg and Marugán-Lobón 2013). Modularity was quantified using a z-transformed covariance ratio (Z-CR) test statistic using the function geomorph:: phylo.modularity() (Adams and Collyer 2019). Further detail on z-score calculation and comparison for modularity hypotheses can be found in Adams and Collyer (2019).

Modularity test results were interpreted in two ways: (1) modularity strength and (2) modularity range. If a modularity hypothesis is stronger or weaker compared to other modularity hypotheses proposed it should show significantly different covariation structure relative to the other modularity hypotheses tested, not just the null hypothesis—we refer to this metric as modularity strength in our results and throughout the remainder of the manuscript. To evaluate this we calculated permuted effect sizes of the Z-CR modularity test statistic for the modularity hypotheses, which were subsequently contrasted in pair-wise comparisons using the function geomorph:: compare.pls().

Modularity range is a metric that captures variation between the upper and lower premolar molar boundary. We collected and analyzed the range of effect sizes across all modularity hypotheses studied for both the upper and lower premolar molar boundary. We define modularity range as the breadth of modularity strength effect size across all modularity hypotheses when compared to a null hypothesis of no modularity. Comparing the modularity range of the upper and lower premolar molar boundary allowed study of the covariation structure between the upper and lower premolar molar boundary.

All code was developed using R version 4.4.2 with the package Geomorph version 4.0.9 (Adams and Otárola-Castillo 2013; R Core Team 2020). All shape files and R code used to complete these analyses are accessioned at the following Dryad Digital Repository 10.5061/dryad.ns1rn8q7k.

## Results

Artiodactyls and perissodactyls each had a significant phylogenetic signal in shape data across both upper and lower premolar molar boundaries which required the use of phylogenetically informed modularity analyses (Artiodactyl Upper K_mult_ = 0.85, *p* < 0.05; Artiodactyl Lower K_mult_ = 1.00, *p* < 0.05; Perissodactyl Upper K_mult_ = 0.89, *p* < 0.05; Perissodactyl Lower K_mult_ = 1.23, *p* < 0.05). Prior to providing our main modularity test results, it is important to note that modularity tests conducted show significant and strong modular support for the Validation hypothesis for both orders at both the upper and lower premolar molar boundary (Artiodactyl Upper ES = –7.21, *p* < 0.05; Artiodactyl Lower ES = –8.04, *p* < 0.05; Perissodactyl Upper ES = –7.05, *p* < 0.05; Perissodactyl Lower ES = –7.11, *p* < 0.05). We are therefore confident in our ability to use this landmark scheme to infer modularity across the premolar molar boundary as a proxy of the PCM.

### Artiodactyls are consistent in overall cusp and cuspid shape covariation structure between the upper and lower premolar-molar boundaries

**Fig. 6:**
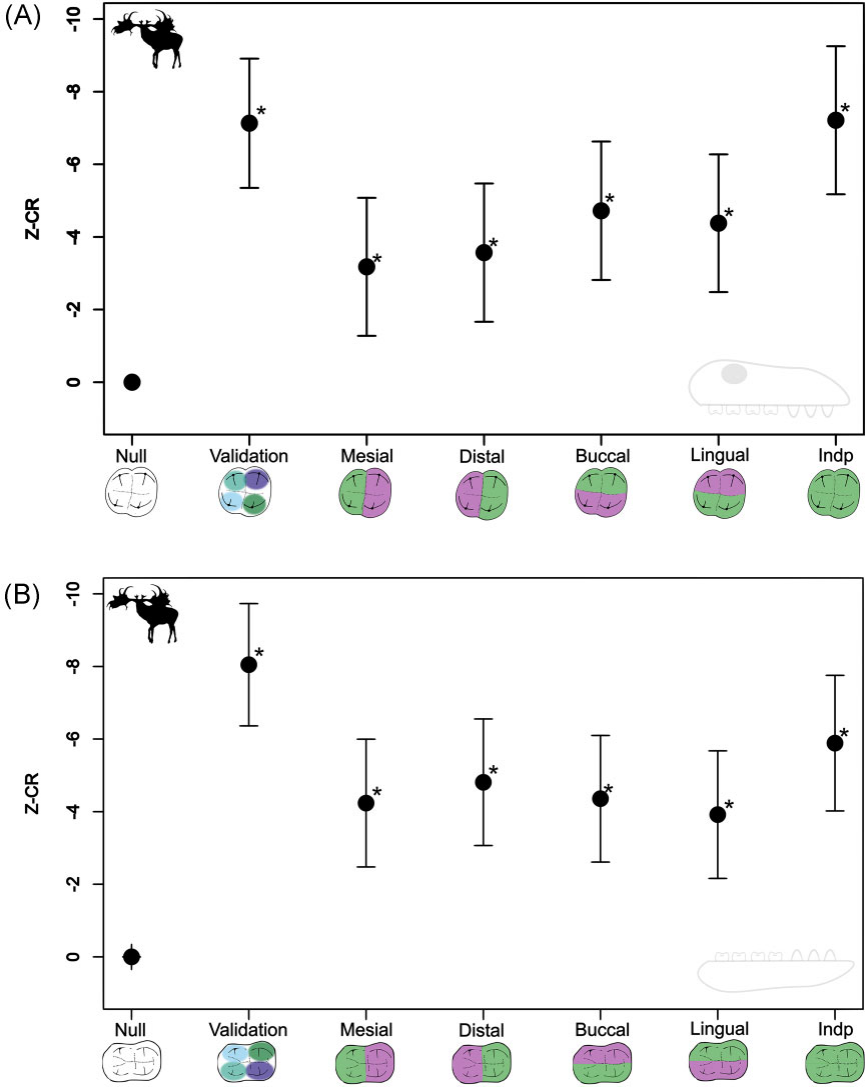
Modularity results for artiodactyls at the (A) upper premolar molar boundary and the (B) lower premolar molar boundary. Asterisks indicate a significant signal of modularity compared to the null of no modularity at a threshold *p*-value of 0.05. More negative Z-CR scores indicate stronger support for a given modularity hypothesis (see methodology). The shaded tooth next to the modularity hypothesis label for each hypothesis represents the premolar patterning (green shading) relative to the molar patterning (purple shading) under that hypothesis.

All upper and lower modularity hypotheses tested in artiodactyls have significant support across the premolar molar boundary (ES > –3.18, *p* < 0.05; Table 1; Fig. 6). The best supported modularity hypothesis at the upper premolar molar boundary is the Independence hypothesis (ES = –7.21) while the least supported is the Mesial hypothesis (ES = –3.18). The best supported modularity hypothesis for the lower premolar molar boundary is the Independence hypothesis (ES = –5.88) while the least supported is the Lingual hypothesis (–3.92). The only difference between the upper and lower premolar molar boundary is in modularity range—the upper premolar molar boundary modularity range is larger than that of the lower premolar molar boundary (4.03 upper boundary; 1.96 lower boundary). While modularity range differs, no significant differences in modularity strength exist between these hypotheses for both the upper and lower premolar molar boundary in artiodactyls (*p* > 0.05; Table 1; Fig. 6).

**Table 1:** Effect sizes for modularity hypothesis pair-wise comparisons of shape at the upper and lower premolar-molar boundary in artiodactyls. Upper (Upp) results are presented to the left of the dotted line while lower (Low) results are presented to the right of the dotted line. Numbers here represent the absolute effect sizes as multivariate standard deviates between observed and expected fit between different modularity hypotheses. Hypothesis comparison proceeded by performing two-sample z-tests that used the pooled standard error from the sampling distributions of the CR analyses. Bold values represent significant differences in effect size for the respective modularity hypothesis comparison at a threshold of *p* < 0.05.

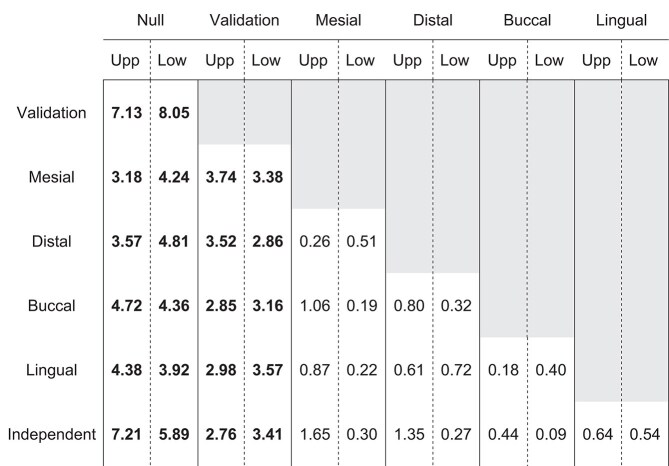

### Perissodactyls are consistent only in some aspects of cusp shape covariation structure between the upper and lower premolar-boundaries

**Fig. 7:**
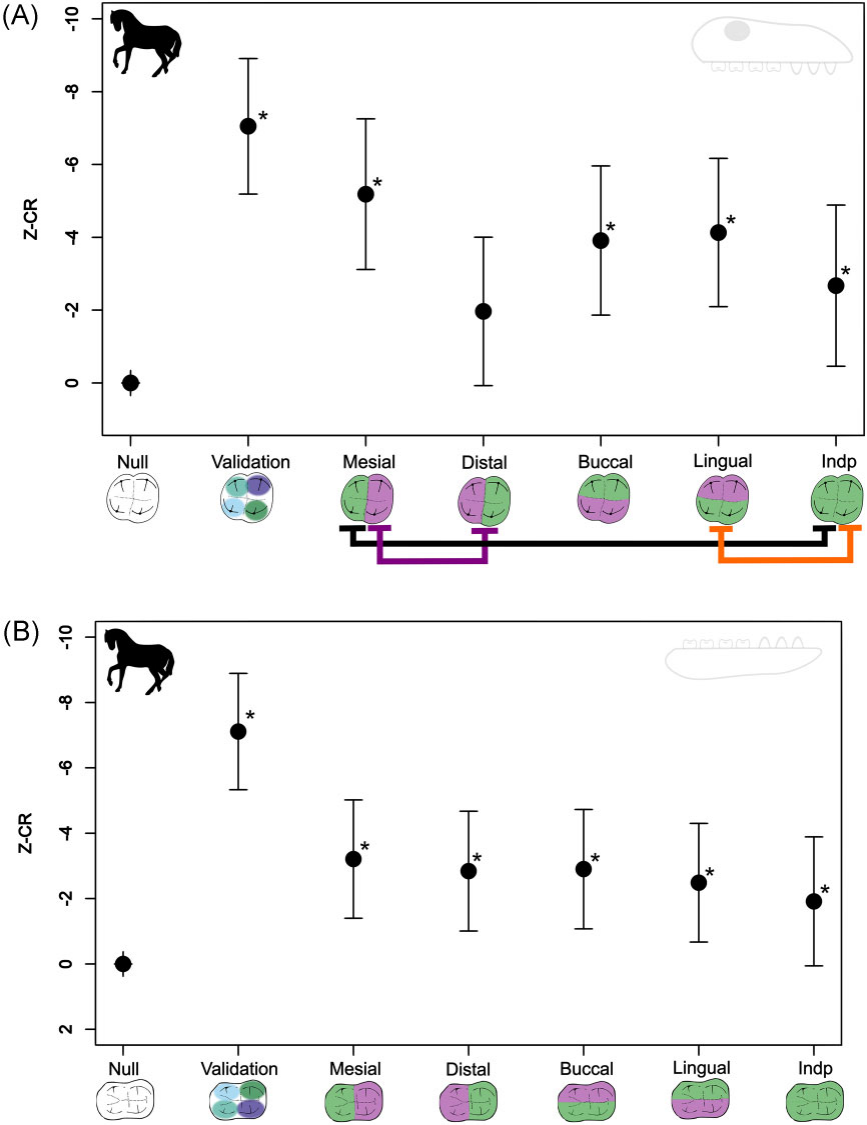
Modularity results for perissodactyls at the (A) upper premolar molar boundary and the (B) lower premolar molar boundary. Asterisks indicate a significant signal of modularity compared to the null of no modularity at a threshold *p*-value of 0.05. More negative Z-CR scores indicate stronger support for a given modularity hypothesis (see Methods). The shaded tooth next to the Z-CR values of each hypothesis represents the premolar patterning (green shading) relative to the molar patterning (purple shading) under that hypothesis. Bars connecting modularity hypotheses indicate a significant difference in modularity signal between the connected hypotheses.

Only one modularity hypothesis, the upper Distal hypothesis, does not have significant modular support for perissodactyls (ES = −1.96, *p* > 0.05; Table 2; Fig. 7A). All other upper and lower modularity hypotheses are significantly supported across the premolar-molar boundary (ES > –1.91, *p* < 0.05; Table 2; Fig. 7). The best supported modularity hypothesis at the upper premolar molar boundary is the Mesial hypothesis (ES = –5.18) while the least supported is the Distal hypothesis (ES = –1.96). The best supported modularity hypothesis at the lower premolar molar boundary is the Mesial hypothesis (ES = –3.21) while the least supported is the Independence hypothesis (–1.91). Similar to artiodactyls, the upper premolar molar boundary modularity range is higher than that of the lower premolar molar boundary modularity range—the modularity range is nearly three times larger at the upper premolar molar boundary compared to the lower premolar molar boundary (3.22 upper boundary; 1.25 lower boundary).

**Table 2:** Effect sizes for modularity hypothesis pair-wise comparisons of shape at the upper and lower premolar-molar boundary in perissodactyls. Upper (Upp) results are presented to the left of the dotted line while perissodactyl (Low) results are presented to the right of the dotted line. Numbers here represent the absolute effect sizes as multivariate standard deviates between observed and expected fit between different modularity hypotheses. Hypothesis comparison proceeded by performing two-sample z-tests that used the pooled standard error from the sampling distributions of the CR analyses. Bold values represent significant differences in effect size for the respective modularity hypothesis comparison at a threshold of *p* < 0.05.

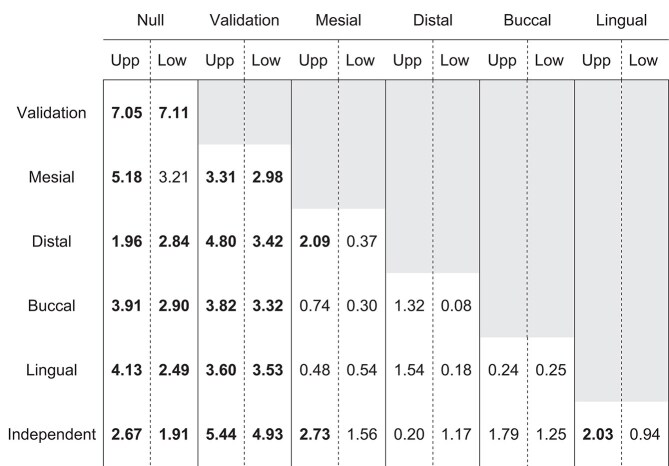

In contrast to artiodactyls, there are two sets of pairwise comparisons of modularity hypotheses across the upper premolar molar boundary perissodactyls that are significantly different in support for modular strength (*p* < 0.05; Table 2; Fig. 7A). First, the Mesial hypothesis is significantly better supported when compared to the Independence hypothesis (ES = 2.73; *p* < 0.05) and the Distal hypothesis (ES = 2.09; *p* < 0.05). Second, the Lingual hypothesis is significantly better supported when compared to the Independence hypothesis (ES = 2.03; *p* < 0.05). While the upper premolar molar boundary illustrates differences in modularity support among hypotheses, no significant differences in modularity strength exist at the lower premolar molar boundary in perissodactyls (*p* > 0.05; Table 2; Fig. 7B).

## Discussion

In this study, we use a 2D GMM landmark scheme to study the evolution of ungulate cusp crown complexity across the upper and lower premolar molar boundary. Our research goals focus on evaluating the covariation in occlusal morphology across the premolar molar boundary when the ICM and PCM are integrated within a single methodological framework. To answer our original research question, we conclude that applying developmental models in an integrated morphometric framework to the upper and lower premolar molar boundary produces both consistent and diverging covariation structures at the premolar molar boundary in ungulates—consistency in occlusal cheek tooth covariation is demonstrated by modularity range while divergence in occlusal cheek tooth covariation is demonstrated by modularity strength. These results show the complex relationship between deep homology, ecological function, and the evolvability of inter-regional crown complexity between mammalian premolars and molars. We explore this relationship through a detailed analysis of our results, a comparative analysis of our results to known herbivore mesowear scoring, and a focal discussion on the independence hypothesis.

### Modularity range is consistent between artiodactyls and perissodactyls while modularity strength is not consistent

All but one of the modularity test results provide support for significant modular structure across the upper and lower premolar molar boundary in ungulates. There is more variation in modularity strength across the upper premolar molar boundary when compared to the lower premolar molar boundary in both artiodactyls and perissodactyls (Tables 1 and 2; Figs. 6 and 7). All artiodactyl modularity test results are consistent with the results of the lower premolar molar boundary in perissodactyls—no significant differences in modularity strength exist for other modularity test results (Tables 1 and 2). The upper premolar molar boundary in perissodactyls shows a lack of support for the Mesial modularity hypothesis as well as significant differences in modularity strength among the Mesial, Distal, Independence, and Lingual hypotheses (Table 2; Fig. 7A). We argue that significant covariation structure across the premolar molar boundary implies that premolars and molars of artiodactyls and perissodactyls are not independent in their cusp and cuspid patterning evolution (Tables 1 and 2; Figs. 6 and 7). We hypothesize that the differences in modularity strength results between artiodactyls and perissodactyls are representative of ecological selective pressures while the consistency in modularity range between artiodactyls and perissodactyls is representative of the deep homology in dental morphogenesis shared between these orders and other tooth bearing heterodont vertebrates. We will return to the importance of modularity range and strength after discussing our results in the context of ecology (mesowear of the upper and lower premolar molar boundary in artiodactyls and perissodactyls) and development (implications of the independence hypothesis performance).

### Mesowear score variation between the upper and lower cheek teeth for artiodactyls and perissodactyls is similar to modularity strength variation recovered in our study

There is a robust body of literature that shows the macroscopic wear patterns of mammalian cheek teeth can be used to infer mammalian dietary ecologies (Mesowear; Kaiser et al. 2000, 2013; Franz-Odendaal and Kaiser 2003; Kaiser and Fortelius 2003). Mesowear has been analyzed by looking at tooth height, convexity, lateral outline, and width in occlusal view (Fraser and Theodor 2013). Cusp or cuspid convexity and lateral outline are commonly applied mesowear variables in browsing ungulates given the importance of occlusal sharpness and breadth in shearing and pulping easily digestible vegetation (most artiodactyls; Franz-Odendaal and Kaiser 2003). Tooth height changes are associated with grazers, as they typically evolve some form of hypsodonty to withstand the grit and/or microscopic properties of calorie poor vegetation they feed on (most perissodactyls; Kaiser et al. 2000). Mesowear has been used to analyze tooth rows but has also yielded important information for grazers and browsers when comparing the upper and lower cheek tooth morphology (Kaiser et al. 2000; Franz-Odendaal and Kaiser 2003; Kaiser and Fortelius 2003).

Artiodactyls show consistency in mesowear between the upper and lower cheek teeth emphasizing the relationship between occlusal morphology and optimal occlusal interactions (Franz-Odendaal and Kaiser 2003). Perissodactyls show inconsistency in mesowear scoring between the upper and lower cheek teeth which is attributed to the evolution of hypsodonty decoupling precise occlusal interactions through their evolution in favour of increased tooth volume (Kaiser and Fortelius 2003; Kaiser et al. 2013). The evolution of hypsodonty prioritizes the height of the cheek teeth over other mesowear variables that the artiodactyl cheek tooth evolutionary trajectory followed (Kaiser et al. 2013).

Another ecological factor that likely contributes to the variation observed in modularity strength is the difference in gastrointestinal physiology between the artiodactyls and perissodactyls. Foregut fermentation is a specialized form of digestion that is found in ruminant artiodactyls, while perissodactyls are hindgut fermenters (Fletcher et al. 2010; Clauss et al. 2023). Hindgut fermenters are under selective pressure to increase mechanical digestion prior to fermentation when compared to foregut fermenters—thus more pressure exists on perissodactyl (hindgut fermenter) cheek tooth morphology when compared to artiodactyls (foregut fermenters; Zhou et al. 2019).

The pattern of variation in modularity strength between the upper and lower premolar molar boundaries of ungulates mirrors this difference in mesowear and gastrointestinal physiology between these groups. Artiodactyl shape data show no difference in modularity strength between the upper and lower premolar molar boundary which can be attributed to tight regulation between the upper and lower cheek teeth to maintain precise occlusion with limited pressure to modify cheek tooth morphology to accommodate foregut fermentation. Perissodactyl shape data highlight a difference in modularity strength between the upper and lower premolar molar boundary illustrating the decoupling effect between mesowear consistency and crown morphology seen in the evolution of hypsodonty and hindgut fermentation (Kaiser et al. 2013). We hypothesize that what we have described here as modularity strength represents the ecological component of cusp shape covariation between the upper and lower premolar molar boundary of ungulates. Furthermore, we hypothesize that these ecological selective pressures caused a release from the canalization between upper and lower cheek tooth morphology development as recorded in artiodactyl modularity range and strength. Future work evaluating early artiodactyl and perissodactyl taxa will be important in evaluating these proposed hypotheses.

### Independence hypothesis modularity results illustrate that the inhibitory cascade model is one of many contributors to the evolution of molariform complexity

In previous work, taxa with molar morphologies that are not predicted by the ICM have been considered outliers rather than products of a broader patterning system from which they diverged (Uller et al. 2018; Lynch 2023; Kenessey et al. 2024; Stenberg et al. 2024; Lafuma et al. 2025). Our work here shows that exceptions to the ICM can be explained when the ICM is considered as a component within the PCM contributing to the evolution of cheek tooth crown complexity.

There is consistency in modularity range of the Independence hypothesis modularity test between artiodactyls and perissodactyls, but modularity strength varies between the two orders showing support for our first prediction of universal support for the independence hypothesis across the premolar molar boundary. Our second prediction concerning the relative results of testing the independence hypothesis is also supported. The Independence hypothesis is weakly supported among the hypotheses tested in perissodactyls while artiodactyl shape data show that the Independence hypothesis is the strongest supported (Tables 1 and 2; Figs. 6 and 7). However, a signal that we did not predict is the relatively weak modularity strength of the Independence hypothesis. The results of our modularity tests show that the signal of independence is not significantly stronger than the results of any other modularity tests grounded in the context of the PCM for artiodactyls (Tables 1 and 2; Figs. 6 and 7). We argue that these results illustrate that the ICM should be considered a component of the PCM in ungulates. Integration of the ICM within the PCM provides the most holistic understanding of the macroevolutionary forces at work in evolving crown complexity in mammalian cheek teeth.

### Synthesis—the covariation structure of cusp morphology when discussed as modularity range and strength can contribute to our understanding of the complex relationship between deep homology, dietary function, and the evolvability of inter-regional dental phenomena

The PCM and ICM are better applied to understanding the evolution of crown complexity in ungulates when they are studied together and applied to both premolars and molars. The study of these models together in this way can capture the product of the interaction between functional and developmental homologies of cheek teeth.

**Fig. 8:**
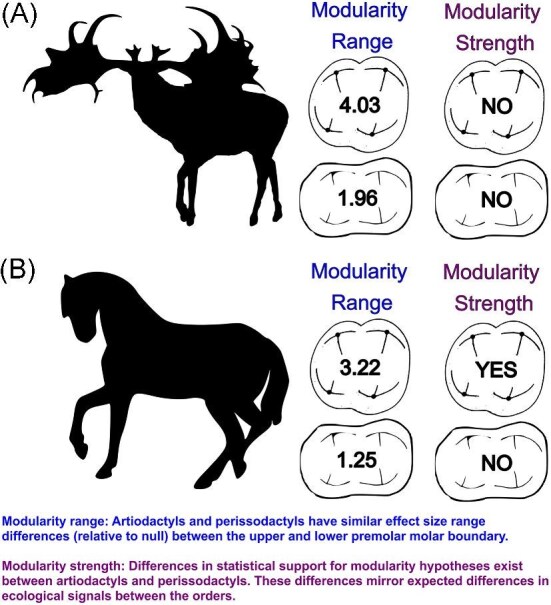
Graphical abstract for (A) artiodactyl and (B) perissodactyl results from our study. Modularity range values depicted show the difference between the largest and smallest effect size calculated between tested modularity hypotheses for the upper (top) or lower (bottom) premolar molar boundary. Modularity strength in this figure indicates whether differences in covariation strength among modularity tests conducted were found (YES) or not (NO; Tables 1, 2).

We hypothesize that the variation in effect size range for modularity tests across the premolar molar boundary is representative of the canalization that has occurred to optimize occlusal function in ungulate cheek teeth (Fig. 8). Cheek tooth morphogenesis initiates with a single primary enamel knot (Jernvall 2000; Jernvall et al. 2000), but functional selective pressures should have a pronounced impact on the spatiotemporal regulation of additional enamel knots that participate in cusp or cuspid morphogenesis. The clearest signal of this canalization in our results exists in effect size range—both orders of ungulates show greater range in modularity strength across the upper premolar molar boundary when compared to the lower premolar molar boundary. We hypothesize that modularity range can be applied as a variable to model the shared evolution of cheek tooth complexity between ungulates. We predict modularity range is a product of the interaction between ancestral mammalian cheek tooth occlusion and the deep developmental homologies at work in modifying cheek tooth morphogenesis.

In contrast to modularity range, we believe that modularity strength is representative of the interactions between occlusal function and the environmental resources available (Fig. 8). Perissodactyl inconsistency and artiodactyl consistency are signals that are consistent with the findings of mesowear studies—artiodactyls show consistency between the upper and lower cheek teeth while perissodactyl upper and lower mesowear scores are decoupled in the evolution of hypsodonty. If modularity strength were modelled in future work, we would predict that it would reflect the relationship between occlusal function, gastrointestinal physiology, and environmental resources.

Cheek tooth occlusion, dietary ecologies, and the developmental homologies of mammals are all tightly interwoven through evolutionary developmental processes—few other groups demonstrate this as clearly as ungulates. The “palimpsest” model of evolutionary-developmental biology is clearly reflected in our results. The integration, de-coupling, canalization, and modularity structures that form and are targeted by selective pressures preclude most methods from evaluating causal relationships in macroevolutionary contexts (Hallgrímsson et al. 2009). Our study serves as an empirical evaluation of how molarization can manifest through eco-functional modules based in developmental context. It is evident that the modularity hypotheses evaluated are not independent of one another but are actually nested within their shared development when the PCM and ICM are considered together. This nested relationship across the upper and lower premolar molar boundary yields important considerations for taxonomic inferences using relative molarization of the premolars in hoofed mammals. Independence of characters is a common methodological assumption for many phylogenetic analyses, and yet there has been an ongoing need to caution against discretizing ordered, meristic characters to fit within the assumptions of taxonomic methods (Brocklehurst and Benevento 2020; Brocklehurst and Haridy 2021). We hope the reader uses the empirical data and results we have discussed here to distill one key take-away—the PCM and ICM are the most informative for studies of cheek tooth macroevolution when they are studied as components of the same theoretical framework rather than mutually exclusive models.

Our results reinforce the importance of framing the PCM and ICM as synergistic actors within the broader processes of macroevolution—dental morphogenesis and its evolution in different dental regions is a product of many independent and cross-talking cascades with every component of these corresponding cascades being a potential target for adaptive and nonadaptive selective pressures alike (Jernvall 2000; Salazar-Ciudad and Jernvall 2002; Ortiz et al. 2018; Damuth and Ginzburg 2025). Our results from this study alongside our previous work illustrate that the premolars and molars share patterning of their cusp or cuspid morphology that can be extended to deep homologies in mammalian dental morphogenesis. There is promise in using the PCM and ICM within a united theoretical framework in studying other instances of cheek tooth evolution including the earlier occurrences of molarization in Eocene perissodactyls prior to the spread of grasslands across North America (Butler 1952). This promise also extends beyond hoofed mammal cheek tooth evolution—carnivorous mammals have an equally long and complicated evolutionary history between their cheek teeth that is worth revisiting under this new framework (Van Valkenburgh 2003, 2007).

We hope this work encourages others to employ the theoretical context of the PCM combined with the methodological framework of modularity analyses to study the evolution of mammalian cheek tooth complexity as a system of dynamic and interacting processes.

### Limitations—similarities and differences in cusp-shape covariation compared to previous work looking solely at the lower cheek teeth

Our previous work is worth revisiting given the current study sampled greater taxonomic breadth as well as the upper and lower dentition compared to only the lower dentition. The improved sampling effort has required us to revise some of our previously proposed hypotheses. However, some signals persist between our studies. The most noteworthy differences between our current results and the previous work concern the Mesial and Independence modularity hypotheses.

The performance of the Mesial (previously the Trigonid hypothesis) and Independence hypotheses is not nearly as strong here for either perissodactyls or artiodactyls as it was in our previous work (Ashbaugh et al. 2025). Using only the lower premolar molar boundary, we showed that artiodactyl shape data favoured the Trigonid hypothesis with the Independence hypothesis as a second alternative (ES_Mesial_ = –4.91, *p* < 0.05; ES_Indp_ = –4.00, *p* < 0.05). For perissodactyls, we failed to reject the null of no modularity for the Trigonid modularity test whereas the strongest hypothesis was the significantly modular Independence hypothesis (ES_Mesial_ = –1.82, *p* > 0.05; ES_Indp_ = –4.55, *p* < 0.05). In the present analysis, the Mesial hypothesis is the best supported modularity hypothesis at the upper and lower premolar molar boundary of perissodactyls while the Independence hypothesis is the strongest performing hypothesis for the upper and lower premolar molar boundary in artiodactyls (Tables 1 and 2; Figs. 6 and 7).

We attribute discrepancies between this study and our previous study to our revised sampling protocol and analytical methods. Our previous study was limited to 18 artiodactyl specimens and 16 perissodactyl specimens, whereas here we have effectively doubled the sample size in all data sets excluding the upper premolar molar boundary of artiodactyls. We have also revised our phylogenetic comparative methods approach allowing for a more accurate representation of species relationships—which strongly impacts the covariation structure in the cusp shape data (Artiodactyl Upper K_mult_ = 0.85, *p* < 0.05; Artiodactyl Lower K_mult_ = 1.00, *p* < 0.05; Perissodactyl Upper K_mult_ = 0.89, *p* < 0.05; Perissodactyl Lower K_mult_ = 1.23, *p* < 0.05).

The performance of the Mesial and Distal hypotheses in our previous work led us to hypothesize that heterochronic shifts were important for the evolution of molarization in artiodactyls. Our results here underscore that heterochronic shifts are one mechanism that likely operates in both artiodactyl and perissodactyl molarization, but they are only one component of this evolutionary process. The performance of the Independence hypothesis here has also led us to revise our previous hypothesis on molarization in perissodactyls. We previously hypothesized that the ICM had a stronger impact on molarization in perissodactyls when compared to artiodactyls. Similar to the reframing in artiodactyls, these independent patterning mechanisms are again one part of a complex evolutionary-developmental program that can lead to molarization in both the upper and lower cheek teeth of ungulates.

## Supplementary Material

obag025_Supplemental_Files
