# Supplementary material for: You Should Look a Gift Ungulate in the Mouth: Using 2D Occlusal Cheek Tooth Morphology to Study the Evolution of Molarization in Ungulates
Source: Integr Org Biol. 2026 May 30;8(1):obag025. doi: 10.1093/iob/obag025 (PMC13266073; doi:10.1093/iob/obag025)
Supplement: obag025_Supplemental_Files [file obag025_supplemental_files.zip › IOB_2026-006_SUPPLEMENTARY FILE_1.docx]

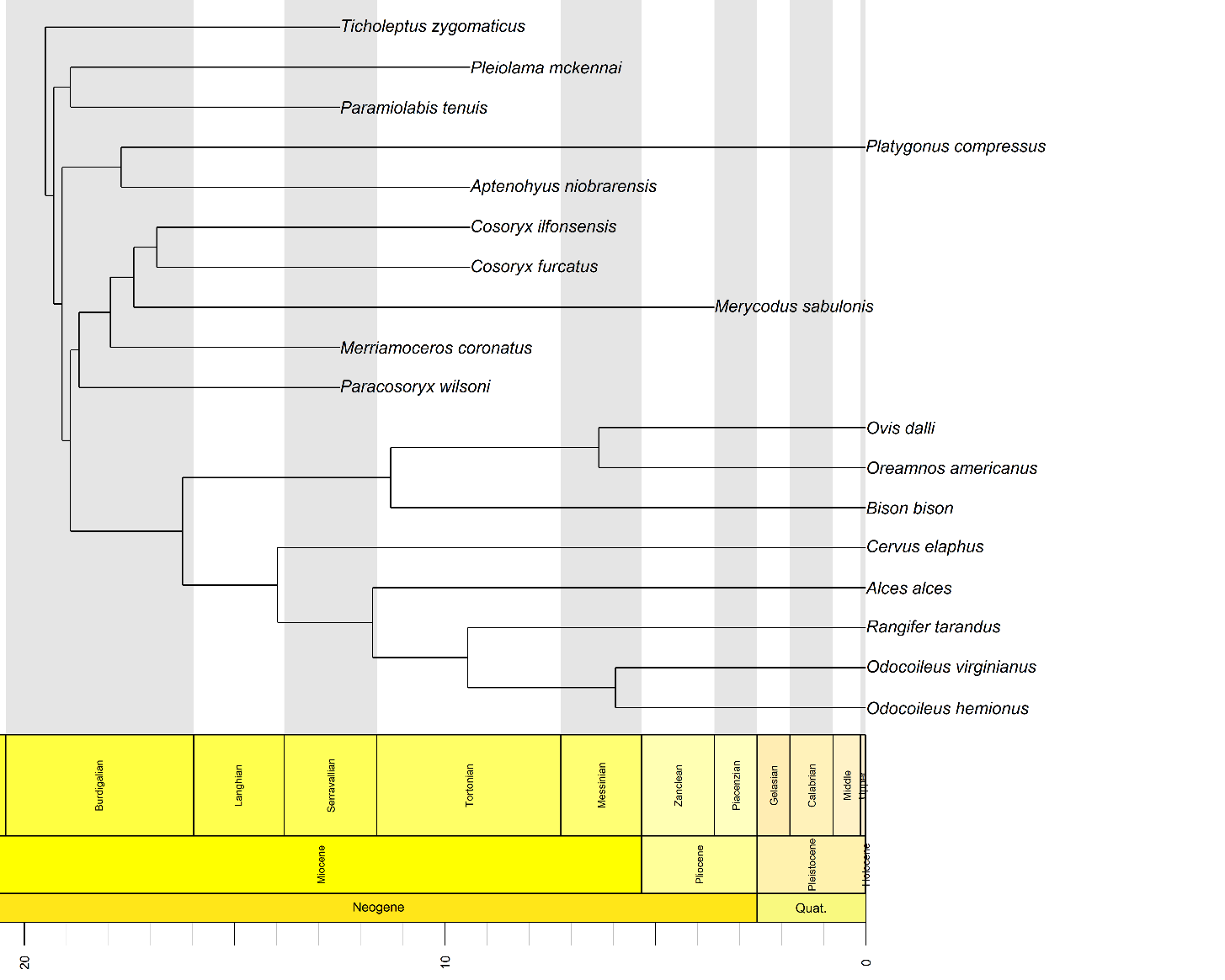


Figure S1.1 Time-scaled phylogenetic tree of all artiodactyl upper premolar molar boundaries imaged. Composite phylogeny based on Fraser at el. (2015) and references therein. Tip-dates were updated according to the paleobiology database (<https://paleobiodb.org/#/>) which are recorded later in this supplementary document.


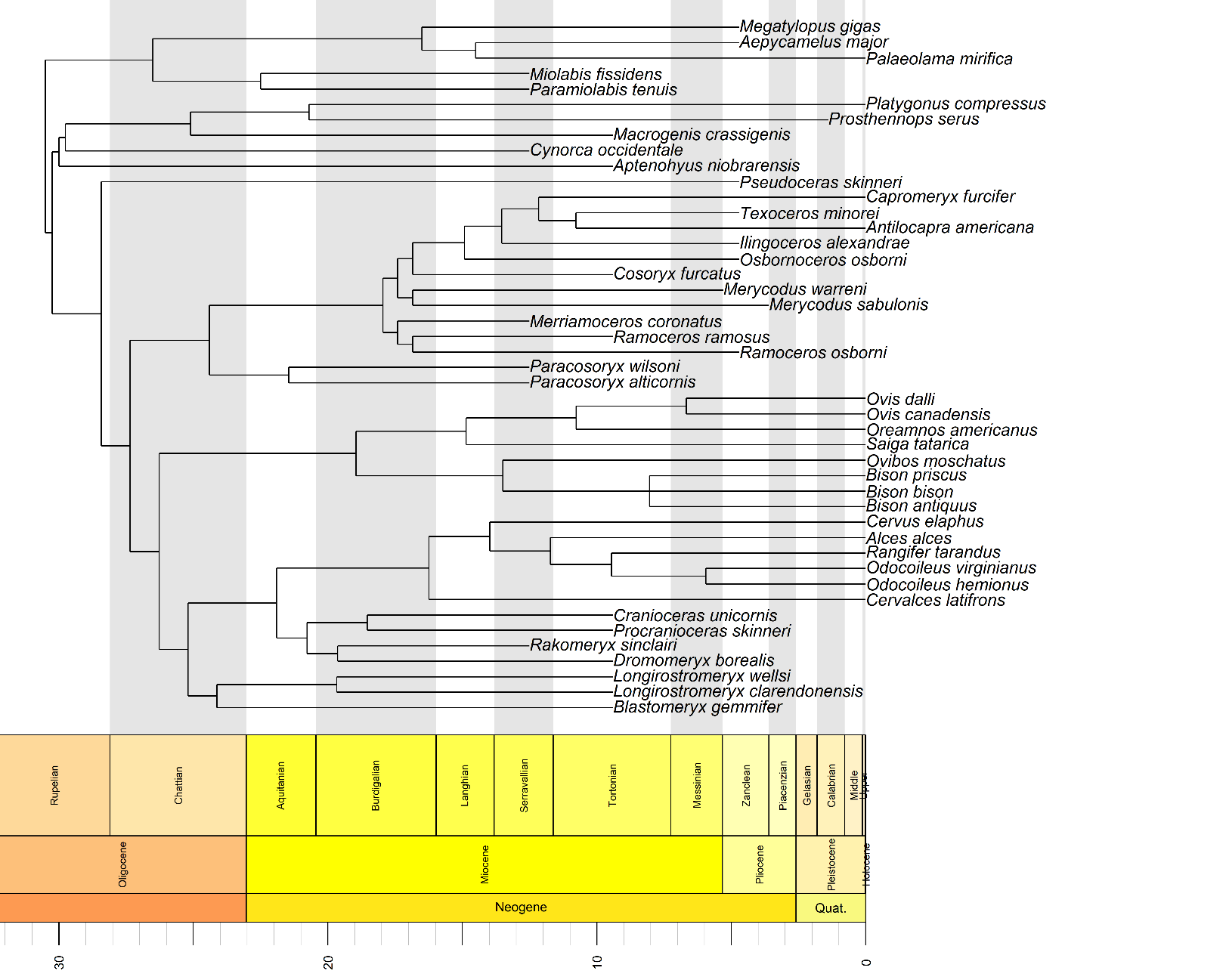


Figure S1.2 Time-scaled phylogenetic tree of all artiodactyl lower premolar molar boundaries imaged. Composite phylogeny based on Fraser at el. (2015) and references therein. Tip-dates were updated according to the paleobiology database (<https://paleobiodb.org/#/>) which are recorded later in this supplementary document.


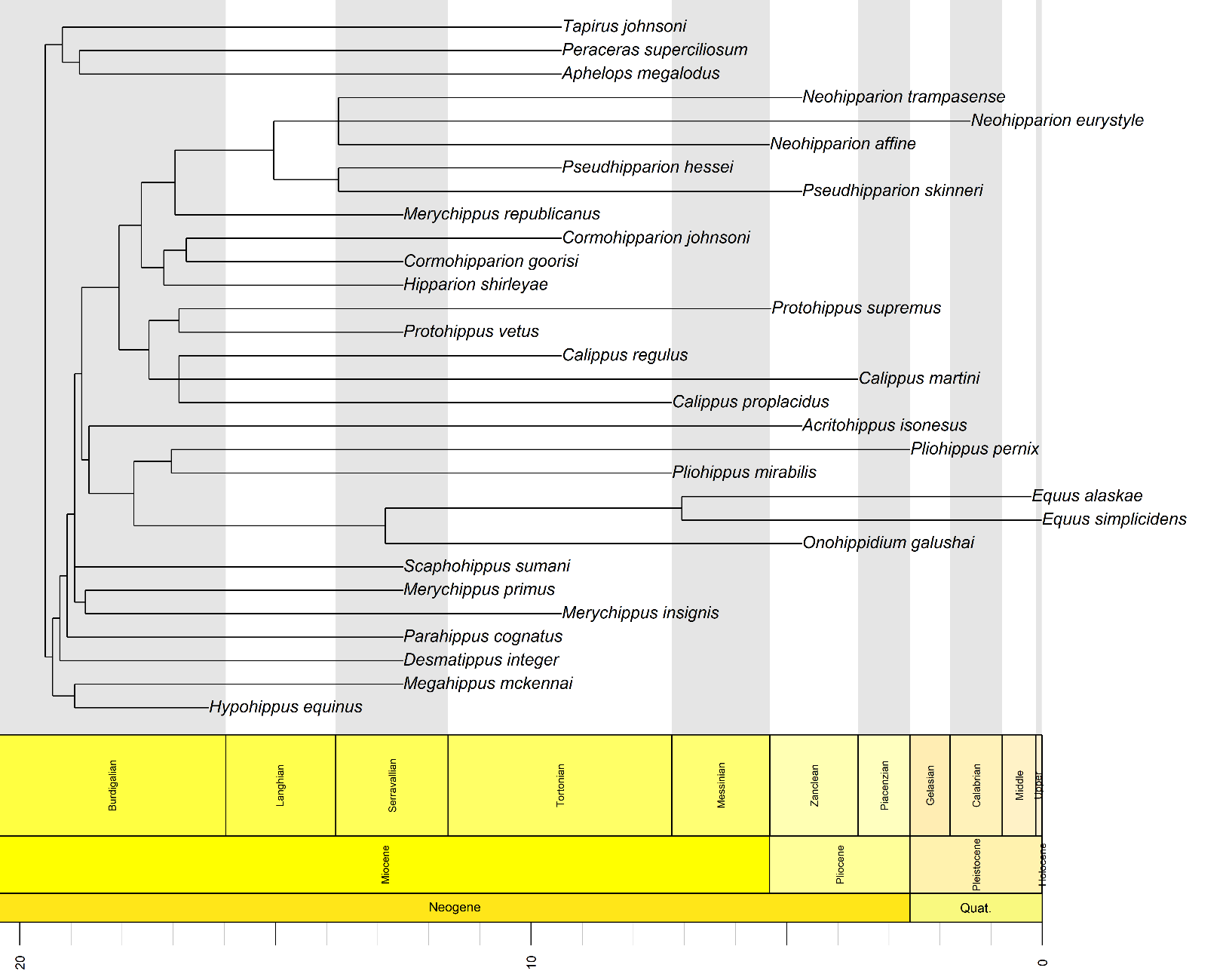


Figure S1.3 Time-scaled phylogenetic tree of all perissodactyl upper premolar molar boundaries imaged. Composite phylogeny based on Fraser at el. (2015) and references therein. Tip-dates were updated according to the paleobiology database (<https://paleobiodb.org/#/>) which are recorded later in this supplementary document.


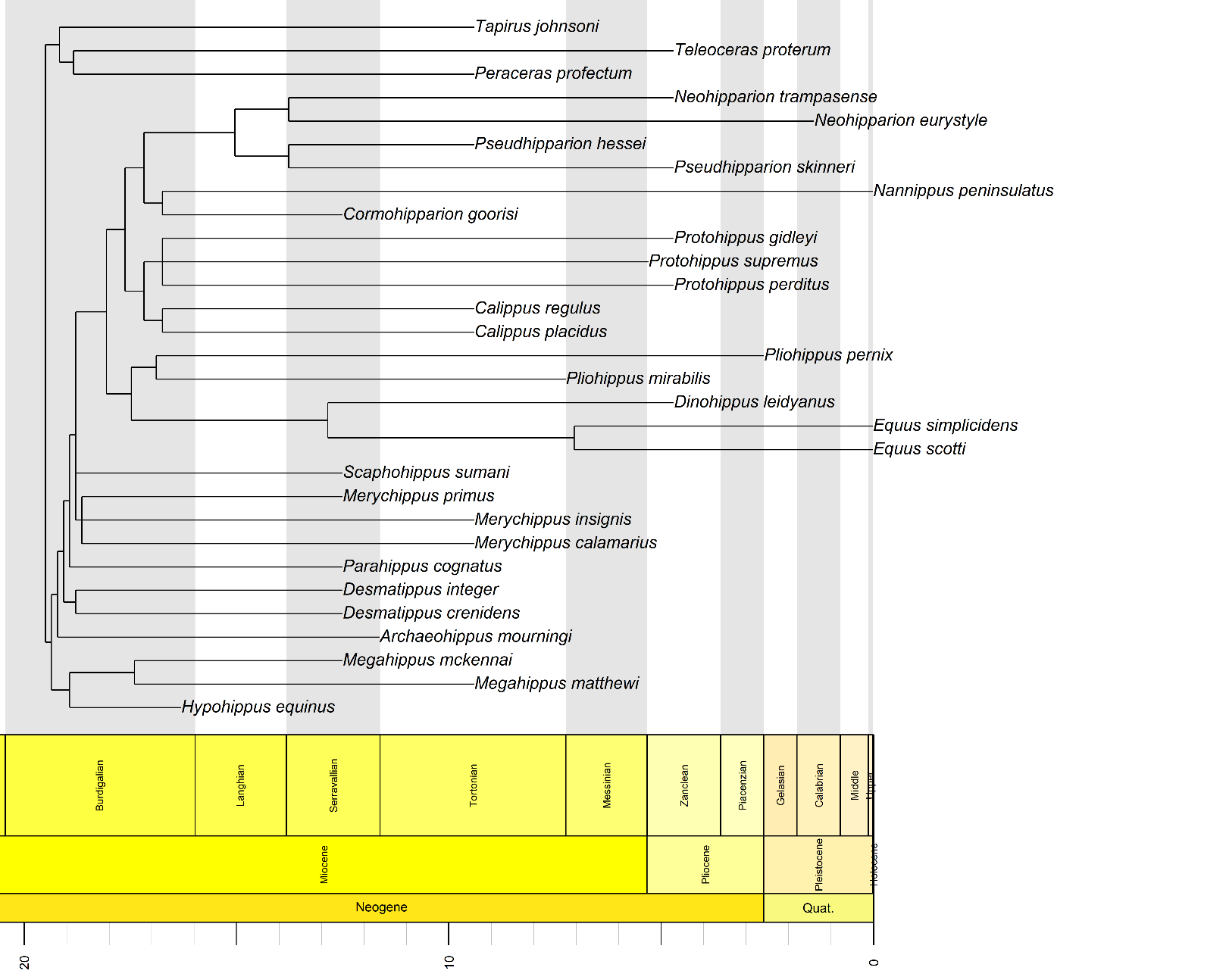


Figure S1.4 Time-scaled phylogenetic tree of all perissodactyl lower premolar molar boundaries imaged. Composite phylogeny based on Fraser at el. (2015) and references therein. Tip-dates were updated according to the paleobiology database (<https://paleobiodb.org/#/>) which are recorded later in this supplementary document.

Nexus file for upper artiodactyls:

#NEXUS

[R-package APE, Fri Aug 22 11:21:12 2025]

BEGIN TAXA;

DIMENSIONS NTAX = 18;

TAXLABELS

Odocoileus_hemionus

Odocoileus_virginianus

Rangifer_tarandus

Alces_alces

Cervus_elaphus

Bison_bison

Oreamnos_americanus

Ovis_dalli

Paracosoryx_wilsoni

Merriamoceros_coronatus

Merycodus_sabulonis

Cosoryx_furcatus

Cosoryx_ilfonsensis

Aptenohyus_niobrarensis

Platygonus_compressus

Paramiolabis_tenuis

Pleiolama_mckennai

Ticholeptus_zygomaticus

;

END;

BEGIN TREES;

TRANSLATE

1 Odocoileus_hemionus,

2 Odocoileus_virginianus,

3 Rangifer_tarandus,

4 Alces_alces,

5 Cervus_elaphus,

6 Bison_bison,

7 Oreamnos_americanus,

8 Ovis_dalli,

9 Paracosoryx_wilsoni,

10 Merriamoceros_coronatus,

11 Merycodus_sabulonis,

12 Cosoryx_furcatus,

13 Cosoryx_ilfonsensis,

14 Aptenohyus_niobrarensis,

15 Platygonus_compressus,

16 Paramiolabis_tenuis,

17 Pleiolama_mckennai,

18 Ticholeptus_zygomaticus

;

TREE * UNTITLED = [&R] (((((((((1:5.95,2:5.95):3.51,3:9.46):2.26,4:11.72):2.26,5:13.98):2.26,(6:11.29333333,(7:6.346666667,8:6.346666667):4.946666667):4.946666667):2.66,(9:6.2,(10:5.45,(11:13.8,(12:7.45,13:7.45):0.55):0.55):0.75)Antilocapridae:0.2):0.2,(14:8.3,15:17.6883)Tayassuidae:1.4):0.2,(16:6.4,17:9.5):0.4):0.2,18:7);

END;

Nexus file for lower artiodactyls:

#NEXUS

[R-package APE, Fri Aug 22 11:21:12 2025]

BEGIN TAXA;

DIMENSIONS NTAX = 45;

TAXLABELS

Blastomeryx_gemmifer

Longirostromeryx_clarendonensis

Longirostromeryx_wellsi

Dromomeryx_borealis

Rakomeryx_sinclairi

Procranioceras_skinneri

Cranioceras_unicornis

Cervalces_latifrons

Odocoileus_hemionus

Odocoileus_virginianus

Rangifer_tarandus

Alces_alces

Cervus_elaphus

Bison_antiquus

Bison_bison

Bison_priscus

Ovibos_moschatus

Saiga_tatarica

Oreamnos_americanus

Ovis_canadensis

Ovis_dalli

Paracosoryx_alticornis

Paracosoryx_wilsoni

Ramoceros_osborni

Ramoceros_ramosus

Merriamoceros_coronatus

Merycodus_sabulonis

Merycodus_warreni

Cosoryx_furcatus

Osbornoceros_osborni

Ilingoceros_alexandrae

Antilocapra_americana

Texoceros_minorei

Capromeryx_furcifer

Pseudoceras_skinneri

Aptenohyus_niobrarensis

Cynorca_occidentale

Macrogenis_crassigenis

Prosthennops_serus

Platygonus_compressus

Paramiolabis_tenuis

Miolabis_fissidens

Palaeolama_mirifica

Aepycamelus_major

Megatylopus_gigas

;

END;

BEGIN TREES;

TRANSLATE

1 Blastomeryx_gemmifer,

2 Longirostromeryx_clarendonensis,

3 Longirostromeryx_wellsi,

4 Dromomeryx_borealis,

5 Rakomeryx_sinclairi,

6 Procranioceras_skinneri,

7 Cranioceras_unicornis,

8 Cervalces_latifrons,

9 Odocoileus_hemionus,

10 Odocoileus_virginianus,

11 Rangifer_tarandus,

12 Alces_alces,

13 Cervus_elaphus,

14 Bison_antiquus,

15 Bison_bison,

16 Bison_priscus,

17 Ovibos_moschatus,

18 Saiga_tatarica,

19 Oreamnos_americanus,

20 Ovis_canadensis,

21 Ovis_dalli,

22 Paracosoryx_alticornis,

23 Paracosoryx_wilsoni,

24 Ramoceros_osborni,

25 Ramoceros_ramosus,

26 Merriamoceros_coronatus,

27 Merycodus_sabulonis,

28 Merycodus_warreni,

29 Cosoryx_furcatus,

30 Osbornoceros_osborni,

31 Ilingoceros_alexandrae,

32 Antilocapra_americana,

33 Texoceros_minorei,

34 Capromeryx_furcifer,

35 Pseudoceras_skinneri,

36 Aptenohyus_niobrarensis,

37 Cynorca_occidentale,

38 Macrogenis_crassigenis,

39 Prosthennops_serus,

40 Platygonus_compressus,

41 Paramiolabis_tenuis,

42 Miolabis_fissidens,

43 Palaeolama_mirifica,

44 Aepycamelus_major,

45 Megatylopus_gigas

;

TREE * UNTITLED = [&R] (((((((1:14.71666667,(2:10.27,3:10.27):4.446666667)Moschidae:1.076666667,(((4:10.235,5:7.135):1.135,(6:9.135,7:9.135):2.235)Palaeomerycidae:1.135,(8:16.2283,((((9:5.95,10:5.95):3.51,11:9.46):2.26,12:11.72):2.26,13:13.98):2.26)Cervidae:5.665):3.288333333):1.076666667,(((14:8.0243,15:8.036,16:8.0243):5.456,17:13.492):5.456,(18:14.856,(19:10.764,(20:6.672,21:6.672):4.092):4.092):4.092):7.322):1.076666667,((22:8.948888889,23:8.948888889):2.948888889,(((24:12.15,25:7.45):0.55,26:4.9):0.55,((27:13.25,28:11.55):0.55,(29:7.45,(30:10.22,(31:8.84,((32:10.78,33:6.08):1.38,34:12.1483):1.38):1.38):1.93):0.55):0.55):6.447777778)Antilocapridae:2.948888889):1.076666667,35:23.72333333):1.826666667,(36:20.6,(37:17.25,(38:15.7,(39:19.3,40:20.6883):4.4):4.65):0.25)Tayassuidae:0.25):0.25,((41:10,42:10):4,((43:14.4883,44:9.8):2,45:11.8):10):4);

END;

Nexus file for upper perissodactyls:

#NEXUS

[R-package APE, Fri Aug 22 11:21:12 2025]

BEGIN TAXA;

DIMENSIONS NTAX = 30;

TAXLABELS

Hypohippus_equinus

Megahippus_mckennai

Desmatippus_integer

Parahippus_cognatus

Merychippus_insignis

Merychippus_primus

Scaphohippus_sumani

Onohippidium_galushai

Equus_simplicidens

Equus_alaskae

Pliohippus_mirabilis

Pliohippus_pernix

Acritohippus_isonesus

Calippus_proplacidus

Calippus_martini

Calippus_regulus

Protohippus_vetus

Protohippus_supremus

Hipparion_shirleyae

Cormohipparion_goorisi

Cormohipparion_johnsoni

Merychippus_republicanus

Pseudhipparion_skinneri

Pseudhipparion_hessei

Neohipparion_affine

Neohipparion_eurystyle

Neohipparion_trampasense

Aphelops_megalodus

Peraceras_superciliosum

Tapirus_johnsoni

;

END;

BEGIN TREES;

TRANSLATE

1 Hypohippus_equinus,

2 Megahippus_mckennai,

3 Desmatippus_integer,

4 Parahippus_cognatus,

5 Merychippus_insignis,

6 Merychippus_primus,

7 Scaphohippus_sumani,

8 Onohippidium_galushai,

9 Equus_simplicidens,

10 Equus_alaskae,

11 Pliohippus_mirabilis,

12 Pliohippus_pernix,

13 Acritohippus_isonesus,

14 Calippus_proplacidus,

15 Calippus_martini,

16 Calippus_regulus,

17 Protohippus_vetus,

18 Protohippus_supremus,

19 Hipparion_shirleyae,

20 Cormohipparion_goorisi,

21 Cormohipparion_johnsoni,

22 Merychippus_republicanus,

23 Pseudhipparion_skinneri,

24 Pseudhipparion_hessei,

25 Neohipparion_affine,

26 Neohipparion_eurystyle,

27 Neohipparion_trampasense,

28 Aphelops_megalodus,

29 Peraceras_superciliosum,

30 Tapirus_johnsoni

;

TREE * UNTITLED = [&R] (((1:2.628571429,2:6.428571429):0.4285714286,(3:6.714285714,(4:6.571428571,((5:9.314285714,6:6.214285714):0.2142857143,7:6.428571429,((((8:8.15,(9:7.0383,10:6.84):5.8):4.916666667,(11:9.787333333,12:14.45333333):0.7333333333):0.8761904762,13:13.94285714)Equini:0.1428571429,(((14:9.640666667,15:13.28666667,16:7.486666667):0.5866666667,(17:4.386666667,18:11.58666667):0.5866666667)Protohippini:0.5866666667,((19:4.68,(20:4.24,21:7.34):0.44):0.44,(22:4.46,((23:9.066666667,24:4.366666667):1.266666667,(25:8.433666667,26:12.36666667,27:9.066666667):1.266666667):1.926666667):0.66)Hipparionini:0.44):0.7257142857):0.1428571429)Equinae:0.1428571429):0.1428571429):0.1428571429):0.1428571429,((28:9.433333333,29:9.433333333):0.3333333333,30:9.766666667):0.3333333333);

END;

Nexus file for lower perissodactyls:

#NEXUS

[R-package APE, Fri Aug 22 11:21:12 2025]

BEGIN TAXA;

DIMENSIONS NTAX = 30;

TAXLABELS

Hypohippus_equinus

Megahippus_matthewi

Megahippus_mckennai

Archaeohippus_mourningi

Desmatippus_crenidens

Desmatippus_integer

Parahippus_cognatus

Merychippus_calamarius

Merychippus_insignis

Merychippus_primus

Scaphohippus_sumani

Equus_scotti

Equus_simplicidens

Dinohippus_leidyanus

Pliohippus_mirabilis

Pliohippus_pernix

Calippus_placidus

Calippus_regulus

Protohippus_perditus

Protohippus_supremus

Protohippus_gidleyi

Cormohipparion_goorisi

Nannippus_peninsulatus

Pseudhipparion_skinneri

Pseudhipparion_hessei

Neohipparion_eurystyle

Neohipparion_trampasense

Peraceras_profectum

Teleoceras_proterum

Tapirus_johnsoni

;

END;

BEGIN TREES;

TRANSLATE

1 Hypohippus_equinus,

2 Megahippus_matthewi,

3 Megahippus_mckennai,

4 Archaeohippus_mourningi,

5 Desmatippus_crenidens,

6 Desmatippus_integer,

7 Parahippus_cognatus,

8 Merychippus_calamarius,

9 Merychippus_insignis,

10 Merychippus_primus,

11 Scaphohippus_sumani,

12 Equus_scotti,

13 Equus_simplicidens,

14 Dinohippus_leidyanus,

15 Pliohippus_mirabilis,

16 Pliohippus_pernix,

17 Calippus_placidus,

18 Calippus_regulus,

19 Protohippus_perditus,

20 Protohippus_supremus,

21 Protohippus_gidleyi,

22 Cormohipparion_goorisi,

23 Nannippus_peninsulatus,

24 Pseudhipparion_skinneri,

25 Pseudhipparion_hessei,

26 Neohipparion_eurystyle,

27 Neohipparion_trampasense,

28 Peraceras_profectum,

29 Teleoceras_proterum,

30 Tapirus_johnsoni

;

TREE * UNTITLED = [&R] (((1:2.628571429,(2:8,3:4.9):1.528571429):0.4285714286,(4:7.584285714,((5:6.285714286,6:6.285714286):0.2857142857,(7:6.428571429,((8:9.242857143,9:9.242857143,10:6.142857143):0.1428571429,11:6.285714286,((((12:7.0383,13:7.0383):5.8,14:8.15):4.623333333,(15:9.640666667,16:14.30666667):0.5866666667):0.5866666667,(((17:7.34,18:7.34):0.44,(19:12.04,20:11.44,21:12.04):0.44)Protohippini:0.44,((22:4.24,23:16.7283):0.44,((24:9.066666667,25:4.366666667):1.266666667,(26:12.36666667,27:9.066666667):1.266666667):2.146666667)Hipparionini:0.44):0.44):0.7257142857)Equinae:0.1428571429):0.1428571429):0.1428571429):0.1428571429):0.1428571429,((28:9.433333333,29:14.13333333):0.3333333333,30:9.766666667):0.3333333333);

END;

Table S1.1 Tip-updating for species included in the upper premolar molar boundary artiodactyls data set as per first and last occurrence data from the PBDB (<https://paleobiodb.org/#/>). Abbreviations: FAD, First appearance date; LAD, Last appearance date.

| *Genus* | *Species* | First appearance (MYA) | FAD | Reference | Last appearance (MYA) | LAD | Reference |
| --- | --- | --- | --- | --- | --- | --- | --- |
| *Ticholeptus* | *zygomaticus* | 18.5-16.3 | 18.5 | W. W. Korth. 1997. A new subfamily of primitive pocket mice (Rodentia, Heteromyidae) from the middle Tertiary of North America. *Paludicola* **1(2)**:33-66 | 16.3-12.5 | 12.5 | J. D. Bryant. 1991. New early Barstovian (middle Miocene) vertebrates from the upper Torreya Formation, eastern Florida panhandle. Journal of Vertebrate Paleontology 11(4):472-489 |
| *Pleiolama* | *Mckennai* | 12.5-9.4 | 12.5 | M. F. Skinner and F. W. Johnson. 1984. Tertiary stratigraphy and the Frick Collection of fossil vertebrates from north-central Nebraska. Bulletin of the American Museum of Natural History **178(3)**:1-368 | 12.5-9.4 | 9.4 | M. F. Skinner and F. W. Johnson. 1984. Tertiary stratigraphy and the Frick Collection of fossil vertebrates from north-central Nebraska. *Bulletin of the American Museum of Natural History* **178(3)**:1-368 |
| *Paramiolabis* | *tenuis* | *18.5-16.3* | 18.5 | S. T. Miller. 1980. Geology and mammalian biostratigraphy of a part of the northern Cady Mountains, Mojave Desert, California. United States Geological Survey Open File Report **80-878**:1-122 | 16.3-12.5 | 12.5 | *. S. Kelly. 1992. New Middle Miocene Camels from the Caliente Formation, Cuyama Valley Badlands, California. PaleoBios* **13(52)**:1-22 |
| *Platygonus* | *compressus* | 2.58-0.0117 | 2.58 | M. K. Frazier. 1977. New Records of Neofiber leonardi (Rodentia: Cricetidae) and the Paleoecology of the Genus. Journal of Mammalogy **58(3)**:368-373 | 0.129-0.0117 | 0.0117 | S. D. Emslie and G. S. Morgan. 1995. Taphonomy of a late Pleistocene carnivore den, Dade County, Florida. In D. W. Steadman and J. I. Mead (eds.), *Late Quaternary Environments and Deep History: A Tribute to Paul Martin* 65-83 |
| *Aptenohyus* | *niobrarensis* | 16.3-12.5 | 16.3 | R. H. Tedford and M. E. Hunter. 1984. Miocene marine-nonmarine correlations, Atlantic and Gulf Coastal Plains, North America. Palaeography, Palaeoclimatology, Palaeoecology 47:129-151 | 12.5-9.4 | 9.4 | M. R. Voorhies. 1990. Vertebrate biostratigraphy of the Ogallala Group in Nebraska. In T. C. Gustavson (ed.), *Geologic Framework and Regional Hydrology: Upper Cenozoic Blackwater Draw and Ogallala Formations, Great Plains* 115-151 |
| *Paracosoryx* | *wilsoni* | 18.5-16.3 | 18.5 | M. F. Skinner, S. M. Skinner, and R. J. Gooris. 1977. Stratigraphy and biostratigraphy of late Cenozoic deposits in central Sioux County, western Nebraska. Bulletin of the American Museum of Natural History **158(5)**:263-370 | 16.3-12.5 | 12.5 | M. F. Skinner, S. M. Skinner, and R. J. Gooris. 1977. Stratigraphy and biostratigraphy of late Cenozoic deposits in central Sioux County, western Nebraska. *Bulletin of the American Museum of Natural History* **158(5)**:263-370 |
| *Merycodus* | *sabulonis* | 11.63-5.333 | 11.63 | Troxell, E. L. (1916). An early Pliocene one-toed horse, Pliohippus lullianus sp. nov. American Journal of Science, 335-348. | 5.333-3.6 | 3.6 | Troxell, E. L. (1916). An early Pliocene one-toed horse, Pliohippus lullianus sp. nov. American Journal of Science, 335-348. |
| *Cosoryx* | *ilfonsensis* | 16.3-12.5 | 16.3 | R. H. Tedford. 1981. Mammalian biochronology of the late Cenozoic basins of New Mexico. Geological Society America Bulletin **92** | 16.3-9.4 | 9.4 | S. G. Lucas and R. M. Schoch. 1982. Duchesneodus, a New Name for Some Titanotheres (Perissodactyla, Brontotheriidae) from the Late Eocene of Western North America. *Journal of Paleontology* **56(4)**:1018-1023 |
| *Cosoryx* | *furcatus* | *16.3-12.5* | 16.3 | X. Wang, R. H. Tedford, and B. E. Taylor. 1999. Phylogenetic systematics of the Borophaginae (Carnivora: Canidae). Bulletin of the American Museum of Natural History **243**:1-392 | 12.5-9.4 | 9.4 | *X. Wang, R. H. Tedford, and B. E. Taylor. 1999. Phylogenetic systematics of the Borophaginae (Carnivora: Canidae). Bulletin of the American Museum of Natural History* **243**:1-392 |
| *Merriamoceros* | *coronatus* | 16.3-12.5 | 16.3 | J. C. Merriam. 1919. Tertiary mammalian faunas of the Mohave desert. University of California Publications, Bulletin of the Department of Geology **11(5)**:437-585 | 16.3-12.5 | 12.5 | J. C. Merriam. 1919. Tertiary mammalian faunas of the Mohave desert. *University of California Publications, Bulletin of the Department of Geology* **11(5)**:437-585 |
| *Ovis* | *dalli* | 0.129-0.0117 | 0.129 | C. R. Harington. 1980. Pleistocene mammals from Lost Chicken Creek, Alaska. Canadian Journal of Earth Sciences 17(2):168-198 | 0 | 0 | Extant |
| *Oreamnos* | *americanus* | 1.4-0.21 | 1.4 | T. S. Kelly. 1995. Current Research in the Pleistocene 12:99-102 | 0 | 0 | Extant |
| *Bison* | *bison* | 0.21-0.014 | 0.21 | C. J. Bell, W. Godwin, K. M. Jenkins and P. J. Lewis. 2021. First fossil manatees in Texas, USA: Trichechus manatus bakerorum from Pleistocene beach deposits along the Gulf of Mexico. *Palaeontologia Electronica* **23(3)**:a47 | 0 | 0 | Extant |
| *Cervus* | *elaphus* | 3.6-0.774 | 3.6 | G. D'Erasmo. 1933. La fauna della grotta di Loretello presso Venosa. *Societa Nazionale di Scienze, Lettere ed Arti* **19(4)**:1-18 | 0 | 0 | Extant |
| *Alces* | *alces* | 2.58 - 0.774 | 2.58 | H.-D. Kahlke. 1958. Die jungpleistozänen Säugetierfaunen aus dem Travertingebiet von Taubach-Weimar-Ehringsdorf (The Early Plestocene mammalian fauna from the travetin-area of Taubach-Weimar-Ehringsdorf). *Alt-Thüringen* **3**:97-130 | 0 | 0 | Extant |
| *Rangifer* | *tarandus* | 7.2 - 0.774 | 7.2 | K. Post and M. Bosselaers. 2017. Cetacean fossils from a 1961 expedition at the Schelde estuary, province of Zeeland, The Netherlands. *Cainozoic Research* **17(1)**:11-21 | 0 | 0 | Extant |
| *Odocoileus* | *virginianus* | 4.7-1.4 | 4.7 | S. D. Webb. 1974. Chronology of Florida Pleistocene mammals. In S. D. Webb (ed.), Pleistocene Mammals of Florida 5-31 | 0 | 0 | Extant |
| *Odocoileus* | *hemionus* | 1.4-0.21 | 1.4 | R. E. Reynolds and W. A. Reeder. 1991. The San Timoteo Formation, Riverside County, California. San Bernardino County Museum Association Quarterly 38(3):44-48 | 0 | 0 | Extant |

Table S1.2 Tip-updating for species included in the lower premolar molar boundary artiodactyls data set as per first and last occurrence data from the PBDB (<https://paleobiodb.org/#/>). Abbreviations: FAD, First appearance date; LAD, Last appearance date.

| *Genus* | *Species* | First appearance (MYA) | FAD | Reference | Last appearance (MYA) | LAD | Reference |
| --- | --- | --- | --- | --- | --- | --- | --- |
| *Megatylopus* | *gigas* | 12.5-9.4 | 12.5 | M. R. Voorhies. 1990. Vertebrate biostratigraphy of the Ogallala Group in Nebraska. In T. C. Gustavson (ed.), *Geologic Framework and Regional Hydrology: Upper Cenozoic Blackwater Draw and Ogallala Formations, Great Plains* 115-151 | 9.4-4.7 | 4.7 | R. A. Rapp. 2006. Owyhee bones: Hemphillian (latest Miocene) Little Jacks Creek (LJC) and Sugar Creek (SC) terrestrial local faunas of the western Snake River Plain, Owyhee County, Idaho. *Idaho State University* i-397 |
| *Aepycamelus* | *major* | 12.5-9.4 | 12.5 | S. D. Webb. 1969. The Burge and Minnechaduza Clarendonian Mammalian Faunas of North-Central Nebraska. *University of California Publications in Geological Sciences* **78**:1-191 | 9.4-4.7 | 4.7 | R. C. Hulbert, A. Poyer, and S. D. Webb. 2002. Tyner Farm, a new early Hemphillian local fauna from north-central Florida. *Journal of Vertebrate Paleontology* **22(3)**:68A |
| *Palaeolama* | *mirifica* | 2.6 - 0.0117 | 2.6 | S.D. Webb and E. Simons. 2006. Vertebrate Paleontology. In S.D. Webb (ed.), *First Floridians and Last Mastodons: The Page-Ladson Site in the Aucilla River* **26**:215-246 | 0.1-0.0117 | 0.0117 | R. C. Hulbert, Jr., G. S. Morgan, and A. Kerner. 2009. Collared peccary (Mammalia, Artiodactyla, Tayassuidae, Pecari) from the late Pleistocene of Florida. In L. B. Albright (ed.), *Papers on Geology, Vertebrate Paleontology, and Biostratigraphy in Honor of Michael O. Woodburne* 543-555 |
| *Miloabis* | *princetonianus* | N/A | N/A | N/A | N/A | N/A | N/A |
| *Miolabis* | *fissidens* | 18.5-16.3 | 18.5 | M. Cassiliano. 1980. Stratigraphy and vertebrate paleontology of the Horse Creek-Trail Creek area, Laramie County, Wyoming. *Contributions to Geology, University of Wyoming* **19(1)**:25-58 | 16.3-12.5 | 12.5 | R. H. Tedford and S. F. Barghoorn. 1993. Neogene stratigraphy and biochronology of the Española Basin, northern New Mexico. *New Mexico Museum of Natural History Bulletin* **2**:159-168 |
| *Paramiolabis* | *tenuis* | 18.5-16.3 | 18.5 | T. Galusha. 1975. Stratigraphy of the Box Butte Formation, Nebraska. *Bulletin of the American Museum of Natural History* **156(1)** | 16.3-12.5 | 12.5 | T. S. Kelly. 1992. New Middle Miocene Camels from the Caliente Formation, Cuyama Valley Badlands, California. *PaleoBios* **13(52)**:1-22 |
| *Platygonus* | *compressus* | 2.58-0.0117 | 2.58 | M. K. Frazier. 1977. New Records of Neofiber leonardi (Rodentia: Cricetidae) and the Paleoecology of the Genus. *Journal of Mammalogy* **58(3)**:368-373 | 0.129-0.0117 | 0.0117 | S. D. Emslie and G. S. Morgan. 1995. Taphonomy of a late Pleistocene carnivore den, Dade County, Florida. In D. W. Steadman and J. I. Mead (eds.), *Late Quaternary Environments and Deep History: A Tribute to Paul Martin* 65-83 |
| *Prosthennops* | *serus* | 16.3-12.5 | 16.3 | W. W. Korth. 2000. Review of Miocene (Hemingfordian to Clarendonian) mylagaulid rodents (Mammalia) from Nebraska. *Annals of Carnegie Museum* **69(4)**:227-280 | 9.4-1.4 | 1.4 | S. C. Wallace and X. Wang. 2004. Two new carnivores from an unusual late Tertiary forest biota in eastern North America. *Nature* **431**:556-559 |
| *Macrogenis* | *crassigenis* | 12.5-9.4 | 12.5 | M. F. Skinner and F. W. Johnson. 1984. Tertiary stratigraphy and the Frick Collection of fossil vertebrates from north-central Nebraska. *Bulletin of the American Museum of Natural History* **178(3)**:1-368 | 12.5-9.4 | 9.4 | J. T. Gregory. 1942. Pliocene Vertebrates From Big Spring Canyon South Dakota. *University of California Publications, Bulletin of the Department of Geological Sciences* **26(4)**:307-446 |
| *Cynorca* | *occidentale* | 29.5-12.5 | 29.5 | B. J. MacFadden, M. X. Kirby, A. Rincon, C. Montes, S. Moron, N. Strong, and C. Jaramillo. 2010. Extinct peccary "Cynorca" occidentale (Tayassuidae, Tayassuinae) from the Miocene of Panama and correlations to North America. *Journal of Paleontology* **84(2)**:288-298 | 16.3-12.5 | 12.5 | F. D. Bode. 1935. The Fauna of the Merychippus Zone, North Coalinga District, California. *Carnegie Institution of Washington Publication* **453(6)**:65-96 |
| *Aptenohyus* | *niobrarensis* | 16.3-12.5 | 16.3 | R. H. Tedford and M. E. Hunter. 1984. Miocene marine-nonmarine correlations, Atlantic and Gulf Coastal Plains, North America. *Palaeography, Palaeoclimatology, Palaeoecology* 47:129-151 | 12.5-9.4 | 9.4 | M. R. Voorhies. 1990. Vertebrate biostratigraphy of the Ogallala Group in Nebraska. In T. C. Gustavson (ed.), *Geologic Framework and Regional Hydrology: Upper Cenozoic Blackwater Draw and Ogallala Formations, Great Plains* 115-151 |
| *Capromeryx* | *furcifer* | 1.4-0.2 | 1.4 | C. W. Hibbard and D. W. Taylor. 1960. Two Late Pleistocene Faunas from Southwestern Kansas. *Contributions from the Museum of Paleontology, University of Michigan* **16(1)**:1-223 | 0.1-0.0117 | 0.0117 | W. W. Dalquest and F. B. Stangl, Jr. 1984. Late Pleistocene and early Recent mammals from Fowlkes Cave, southern Culberson County, Texas. *Carnegie Museum of Natural History Special Publication* **8**:432-455 |
| *Texoceros* | *minorei* | 9.4-4.7 | 9.4 | C. J. Hesse. 1936. A Pliocene Vertebrate Fauna From Optima, Oklahoma. *University of California Publications, Bulletin of the Department of Geological Sciences* **24(3)**:57-70 | 9.4-4.7 | 4.7 | B. J. MacFadden, N. M. Johnson, and N. D. Opdyke. 1979. Magnetic polarity stratigraphy of the Mio-Pliocene mammal-bearing Big Sandy Formation of western Arizona. *Earth and Planetary Science Letters* **44(3)**:349-364 |
| *Ilingoceros* | *alexandrae* | 9.4-4.7 | 9.4 | R. A. Stirton. 1940. The Nevada Miocene and Pliocene mammalian faunas as faunal units. *Proceedings of the Sixth Pacific Science Congress* **2**:627-640 | 9.4-4.7 | 4.7 | R. A. Stirton. 1940. The Nevada Miocene and Pliocene mammalian faunas as faunal units. *Proceedings of the Sixth Pacific Science Congress* **2**:627-640 |
| *Osbornoceros* | *osborni* | 9.4-4.7 | 9.4 | R. P. Lozinsky and R. H. Tedford. 1991. Geology and Paleontology of the Santa Fe Group, Southwestern Albuquerque Basin, Valencia County, New Mexico. *Bulletin of the New Mexico Bureau of Mines and Mineral Research* **132**:1-35 | 9.4-4.7 | 4.7 | R. P. Lozinsky and R. H. Tedford. 1991. Geology and Paleontology of the Santa Fe Group, Southwestern Albuquerque Basin, Valencia County, New Mexico. *Bulletin of the New Mexico Bureau of Mines and Mineral Research* **132**:1-35 |
| *Paracosoryx* | *wilsoni* | 18.5-16.3 | 18.5 | M. F. Skinner, S. M. Skinner, and R. J. Gooris. 1977. Stratigraphy and biostratigraphy of late Cenozoic deposits in central Sioux County, western Nebraska. *Bulletin of the American Museum of Natural History* **158(5)**:263-370 | 16.3-12.5 | 12.5 | M. F. Skinner, S. M. Skinner, and R. J. Gooris. 1977. Stratigraphy and biostratigraphy of late Cenozoic deposits in central Sioux County, western Nebraska. *Bulletin of the American Museum of Natural History* **158(5)**:263-370 |
| *Paracosoryx* | *alticornis* | 16.3-12.5 | 16.3 | . E. Storer. 1971. The Wood Mountain fauna: an upper Miocene mammalian assemblage from southern Saskatchewan. **1(513)** | 16.3-12.5 | 12.5 | X. Wang, R. H. Tedford, and B. E. Taylor. 1999. Phylogenetic systematics of the Borophaginae (Carnivora: Canidae). *Bulletin of the American Museum of Natural History* **243**:1-392 |
| *Merycodus* | *warreni* | 16.3-12.5 | 16.3 | M. R. Voorhies. 1971. Paleoclimatic significance of crocodilian remains from the Ogallala Group (upper Tertiary) in northeastern Nebraska. *Journal of Paleontology* **45(1)**:119-121 | 11.6-5.3 | 5.3 | J. G. Honey and G. A. Izett. 1988. Paleontology, taphonomy, and stratigraphy of the Browns Park Formation (Oligocene and Miocene) near Maybell, Moffat County, Colorado. *United States Geological Survey Professional Paper* **1358**:1-52 |
| *Merycodus* | *sabulonis* | 11.63-5.333 | 11.63 | Troxell, E. L. (1916). An early Pliocene one-toed horse, Pliohippus lullianus sp. nov. American Journal of Science, 335-348. | 5.333-3.6 | 3.6 | Troxell, E. L. (1916). An early Pliocene one-toed horse, Pliohippus lullianus sp. nov. American Journal of Science, 335-348. |
| *Cosoryx* | *furcatus* | 16.3-12.5 | 16.3 | X. Wang, R. H. Tedford, and B. E. Taylor. 1999. Phylogenetic systematics of the Borophaginae (Carnivora: Canidae). *Bulletin of the American Museum of Natural History* **243**:1-392 | 12.5-9.4 | 9.4 | X. Wang, R. H. Tedford, and B. E. Taylor. 1999. Phylogenetic systematics of the Borophaginae (Carnivora: Canidae). *Bulletin of the American Museum of Natural History* **243**:1-392 |
| *Merriamoceros* | *coronatus* | 16.3-12.5 | 16.3 | J. C. Merriam. 1919. Tertiary mammalian faunas of the Mohave desert. *University of California Publications, Bulletin of the Department of Geology* **11(5)**:437-585 | 16.3-12.5 | 12.5 | J. C. Merriam. 1919. Tertiary mammalian faunas of the Mohave desert. *University of California Publications, Bulletin of the Department of Geology* **11(5)**:437-585 |
| *Ramoceros* | *ramosus* | 16.3-12.5 | 16.3 | R. H. Tedford. 1981. Mammalian biochronology of the late Cenozoic basins of New Mexico. *Geological Society America Bulletin* **92** | 12.5-9.4 | 9.4 | Anonymous. 1940. The seventh quarterly report covering the quarter ending December 31, 1940 for the state-wide paleontologic-mineralogic survey in Texas. *A Federal Works Agency Work Projects Administration Project. O.P. No. 665-66-3-233. State Serial No. 300-88* 1-44 |
| *Ovis* | *dalli* | 0.129-0.0117 | 0.129 | C. R. Harington. 1980. Pleistocene mammals from Lost Chicken Creek, Alaska. Canadian Journal of Earth Sciences 17(2):168-198 | 0 | 0 | Extant |
| *Ovis* | *canadensis* | 2.58-0.0117 | 2.58 | C. S. Churcher. 1968. Pleistocene ungulates from the Bow River gravels at Cochrane, Alberta. *Canadian Journal of Earth Sciences* 5:1467-1488 | 0 | 0 | Extant |
| *Oreamnos* | *americanus* | 1.4-0.21 | 1.4 | T. S. Kelly. 1995. Current Research in the Pleistocene 12:99-102 | 0 | 0 | Extant |
| *Saiga* | *tatarica* | 0.774-0.129 | 0.774 | B. S. Kozhamkulova. 1986. The Late Cenozoic two-humped (Bactrian) camels of Asia. *Quatarpalaontologie* **6**:93-97 | 0 | 0 | Extant |
| *Ovibos* | *moschatus* | 1.4-0.0117 | 1.4 | P. Remeika. 1992. *San Bernardino County Museum Association Quarterly* **39(1)** | 0 | 0 | Extant |
| *Bison* | *bison* | 0.21-0.014 | 0.21 | C. J. Bell, W. Godwin, K. M. Jenkins and P. J. Lewis. 2021. First fossil manatees in Texas, USA: Trichechus manatus bakerorum from Pleistocene beach deposits along the Gulf of Mexico. *Palaeontologia Electronica* **23(3)**:a47 | 0 | 0 | Extant |
| *Bison* | *antiquus* | 2.58-0.0117 | 2.58 | R. Carrillo-López, A. Velasco-Rodríguez, R. Vásquez-Simon, G. Valera-Venegas, and E. Jiménez-Hidalgo. 2024. New records of Bison (Mammalia: Bovidae) from Southern Mexico and some comments on their distribution and biochronology. *PalZ* **98(1)**:145-159 | 2.58-0.0117 | 0.0117 | D. B. Jones and L. R. G. Desantis. 2017. Dietary ecology of ungulates from the La Brea tar pits in southern California: A multi-proxy approach. Palaeogeography, Palaeoclimatology, Palaeoecology 465(15):110-127 |
| *Cervus* | *elaphus* | 3.6-0.774 | 3.6 | G. D'Erasmo. 1933. La fauna della grotta di Loretello presso Venosa. *Societa Nazionale di Scienze, Lettere ed Arti* **19(4)**:1-18 | 0 | 0 | Extant |
| *Alces* | *alces* | 2.58 - 0.774 | 2.58 | H.-D. Kahlke. 1958. Die jungpleistozänen Säugetierfaunen aus dem Travertingebiet von Taubach-Weimar-Ehringsdorf (The Early Plestocene mammalian fauna from the travetin-area of Taubach-Weimar-Ehringsdorf). *Alt-Thüringen* **3**:97-130 | 0 | 0 | Extant |
| *Rangifer* | *tarandus* | 7.2 - 0.774 | 7.2 | K. Post and M. Bosselaers. 2017. Cetacean fossils from a 1961 expedition at the Schelde estuary, province of Zeeland, The Netherlands. *Cainozoic Research* **17(1)**:11-21 | 0 | 0 | Extant |
| *Odocoileus* | *virginianus* | 4.7-1.4 | 4.7 | S. D. Webb. 1974. Chronology of Florida Pleistocene mammals. In S. D. Webb (ed.), Pleistocene Mammals of Florida 5-31 | 0 | 0 | Extant |
| *Odocoileus* | *hemionus* | 1.4-0.21 | 1.4 | R. E. Reynolds and W. A. Reeder. 1991. The San Timoteo Formation, Riverside County, California. San Bernardino County Museum Association Quarterly 38(3):44-48 | 0 | 0 | Extant |
| *Cranioceras* | *unicornis* | 16.3-12.5 | 16.3 | M. F. Skinner, S. M. Skinner, and R. J. Gooris. 1977. Stratigraphy and biostratigraphy of late Cenozoic deposits in central Sioux County, western Nebraska. *Bulletin of the American Museum of Natural History* **158(5)**:263-370 | 12.5-9.4 | 9.4 | M. R. Voorhies. 1990. Vertebrate biostratigraphy of the Ogallala Group in Nebraska. In T. C. Gustavson (ed.), *Geologic Framework and Regional Hydrology: Upper Cenozoic Blackwater Draw and Ogallala Formations, Great Plains* 115-151 |
| *Rakomeryx* | *sinclairi* | 18.5-16.3 | 18.5 | W. N. F. McLaughlin, S. S. B. Hopkins, and M. D. Schmitz. 2016. A new late Hemingfordian vertebrate fauna from Hawk Rim, Oregon, with implications for biostratigraphy and geochronology. Journal of Vertebrate Paleontology e1201095 :1-21 | 16.3-12.5 | 12.5 | M. F. Skinner, S. M. Skinner, and R. J. Gooris. 1977. Stratigraphy and biostratigraphy of late Cenozoic deposits in central Sioux County, western Nebraska. Bulletin of the American Museum of Natural History 158(5):263-370 |
| *Dromomeryx* | *borealis* | 18.5-16.3 | 18.5 | C. Frick. 1937. Horned ruminants of North America. *Bulletin of the American Museum of Natural History* **69**:1-669 | 12.5-9.4 | 9.4 | P. C. Henshaw. 1939. A Tertiary Mammalian Fauna From the Avawatz Mountains, San Bernardino County, California. Carnegie Institution of Washington Publication 514(1):1-30 |
| *Longirostromeryx* | *wellsi* | 16.3-12.5 | 16.3 | R. H. Tedford. 1981. Mammalian biochronology of the late Cenozoic basins of New Mexico. Geological Society America Bulletin 92 | 12.5-9.4 | 9.4 | M. R. Voorhies. 1990. Vertebrate biostratigraphy of the Ogallala Group in Nebraska. In T. C. Gustavson (ed.), *Geologic Framework and Regional Hydrology: Upper Cenozoic Blackwater Draw and Ogallala Formations, Great Plains* 115-151 |
| *Longirostromeryx* | *clarendonensis* | 12.5-9.4 | 12.5 | R. C. Hulbert, Jr. 1989. Phylogenetic Interrelationships and Evolution of North American Late Equidae. In D. R. Prothero and R. M. Schoch (eds.), *The Evolution of Perissodactyls* | 12.5-9.4 | 9.4 | R. C. Hulbert, Jr. 1989. Phylogenetic Interrelationships and Evolution of North American Late Equidae. In D. R. Prothero and R. M. Schoch (eds.), *The Evolution of Perissodactyls* |
| *Blastomeryx* | *gemmifer* | 23.04-5.333 | 23.04 | T. Downs. 1956. The Mascall fauna from the Miocene of Oregon. *University of California Publications in Geological Sciences* **31(5)**:199-354 | 12.5-9.4 | 9.4 | S. D. Webb. 1969. The Burge and Minnechaduza Clarendonian Mammalian Faunas of North-Central Nebraska. University of California Publications in Geological Sciences 78:1-191 |
| *Antilocapra* | *americana* | 1.4-0.21 | 1.4 | J. I. Mead, C. Manganaro, C. A. Repenning and L. D. Agenbroad. 1996. Early Rancholabrean mammals from Salamander Cave, Black Hills, South Dakota. In K. M. Stewart, K. L. Seymour (eds.), *Palaeoecology and palaeoenvironments of late Cenozoic mammals: Tributes to the career of C.S. (Rufus) Churcher* 458-482 | 0 | 0 | Extant |
| *Ovis* | *aries* | 2.58-0.774 | 2.58 | D. J. Scager, H.-J. Ahrens, F. E. Dieleman, L. W. Hoek Ostende, J. Vos and J. W. F. Reumer. 2017. The Kor & Bot collection revisited, with a biostratigraphic interpretation of the Early Pleistocene Oosterschelde Fauna (Oosterschelde Estuary, the Netherlands). *Deinsea* **17**:16-31 | 0 | 0 | Extant |
| *Procamelus* | *grandis* | 16.3-12.5 | 16.3 | M. R. Voorhies. 1969. Taphonomy and population dynamics of an Early Pliocene vertebrate fauna, Know County, Nebraska. *University of Wyoming Contributions to Geology Special Paper* **1**:1-69 | 12.5-9.4 | 9.4 | M. Cassiliano. 1980. Stratigraphy and vertebrate paleontology of the Horse Creek-Trail Creek area, Laramie County, Wyoming. *Contributions to Geology, University of Wyoming* **19(1)**:25-58 |
| *Pseudoceras* | *skinneri* | 12.5-9.4 | 12.5 | . H. Sellards. 1941. Final report covering the period from March 4, 1939 ro September 30, 1941 for the state-wide paleontologic-mineralogic survey in Texas. *A Federal Works Agency Work Projects Administration Project* iii-85 | 9.4-4.7 | 4.7 | G. E. Schultz. 1990. Stop 15: Early Hemphillian faunas of the Texas and Oklahoma panhandles. In T. C. Gustavson (ed.), *Tertiary and Quaternary stratigraphy and vertebrate paleontology of parts of northwestern Texas and eastern New Mexico; Guidebook - Bureau of Economic Geology, University of Texas at Austin* 95-103 |
| *Pediomeryx* | *hemiphillensis* | 9.4-4.7 | 9.4 | C. J. Hesse. 1936. A Pliocene Vertebrate Fauna From Optima, Oklahoma. *University of California Publications, Bulletin of the Department of Geological Sciences* **24(3)**:57-70 | 9.4-4.7 | 4.7 | M. F. Skinner, S. M. Skinner, and R. J. Gooris. 1977. Stratigraphy and biostratigraphy of late Cenozoic deposits in central Sioux County, western Nebraska. *Bulletin of the American Museum of Natural History* **158(5)**:263-370 |
| *Paratoceras* | *wardi* | 18.5-16.3 | 18.5 | B. J. MacFadden. 2006. North American Miocene Land Mammals from Panama. *Journal of Vertebrate Paleontology* **26(3)**:720-734 | 16.3-12.5 | 12.5 | R. H. Tedford, T. Galusha, M. F. Skinner, B. E. Taylor, R. W. Fields, J. R. Macdonald, J. M. Rensberger, S. D. Webb, and D. P. Whistler. 1987. Faunal succession and biochronology of the Arikareean through Hemphillian interval (late Oligocene through earliest Pliocene epochs) in North America. In M. O. Woodburne (ed.), *Cenozoic Mammals of North America: Geochronology and Biostratigraphy* 153-210 |
| *Cervalces* | *latifrons* | 2.58-0.774 | 2.58 | . von Koenigswald and W.-D. Heinrich. 1999. Mittelpleistozane Saugetierfaunen aus Mitteleuropa - der Versuch einer biostratigraphischen Zuordnung. *Kaupia* **9**:53-112 | 0.129-0.0117 | 0.0117 | M. M. Leighton. 1921. The Pleistocene succession near Alton, Illinois, and the age of the mammalian fossil fauna. *Journal of Geology* **29(6)**:505-514 |
| *Bison* | *priscus* | 2.58-0.0117 | 2.58 | I. M. Cowan. 1941. Fossil and subfossil mammals from the Quaternary of British Columbia. *Transactions of the Royal Society of Canada, Section IV Geological Sciences including Mineralogy, Third Series* **33(4)**:39-50 | 0.129-0.0117 | 0.0117 | C. R. Harington. 1990. Ice Age Vertebrates in the Canadian Arctic Islands. *Canada's Missing Dimension* **I**:140-160 |
| *Merycodus* | *necatus* | 16.3-12.5 | 16.3 | M. R. Voorhies. 1990. Vertebrate paleontology of the proposed Norden Reservoir Area, Brown, Cherry and Keya Paha counties, Nebraska. *Technical Report, Division of Archeological Research, Department of Anthropology, University of Nebraska* **82-09** | 16.3-12.5 | 12.5 | J. F. Sutton and W. W. Korth. 1995. Rodents (Mammalia) from the Barstovian (Miocene) Anceney local fauna, Montana. *Annals of Carnegie Museum* **64(4)**:267-314 |
| *Ramoceros* | *osborni* | 16.3-12.5 | 16.3 | M. R. Voorhies. 1990. Vertebrate biostratigraphy of the Ogallala Group in Nebraska. In T. C. Gustavson (ed.), *Geologic Framework and Regional Hydrology: Upper Cenozoic Blackwater Draw and Ogallala Formations, Great Plains* 115-151 | 9.4-4.7 | 4.7 | C. Frick. 1937. Horned ruminants of North America. *Bulletin of the American Museum of Natural History* **69**:1-669 |
| *Procranioceras* | *skinneri* | 16.3-12.5 | 16.3 | M. R. Voorhies. 1990. Vertebrate paleontology of the proposed Norden Reservoir Area, Brown, Cherry and Keya Paha counties, Nebraska. *Technical Report, Division of Archeological Research, Department of Anthropology, University of Nebraska* **82-09** | 16.3-9.4 | 9.4 | M. R. Voorhies. 1990. Vertebrate paleontology of the proposed Norden Reservoir Area, Brown, Cherry and Keya Paha counties, Nebraska. *Technical Report, Division of Archeological Research, Department of Anthropology, University of Nebraska* **82-09** |

Table S1.3 Tip-updating for species included in the upper premolar molar boundary artiodactyls data set as per first and last occurrence data from the PBDB (<https://paleobiodb.org/#/>). Abbreviations: FAD, First appearance date; LAD, Last appearance date.

| *Genus* | *Species* | First appearance (MYA) | FAD | Reference | Last appearance (MYA) | LAD | Reference |
| --- | --- | --- | --- | --- | --- | --- | --- |
| *Tapirus* | *johnsoni* | 12.5-9.4 | 12.5 | M. A. Turner. 1972. A faunal assemblage from the Lower Ash Hollow Formation (Neogene) of southern Nebraska. *M. S. thesis University of Nebraska* 1-88 | 12.5-9.4 | 9.4 | M. A. Turner. 1972. A faunal assemblage from the Lower Ash Hollow Formation (Neogene) of southern Nebraska. *M. S. thesis University of Nebraska* 1-88 |
| *Peraceras* | *superciliosum* | 16.3-9.4 | 16.3 | W. D. Matthew. 1918. Contributions to the Snake Creek Fauna with notes upon the Pleistocene of western Nebraska, American Museum Expedition of 1916. *Bulletin of the American Museum of Natural History* **38(7)**:183-229 | 16.3-9.4 | 9.4 | W. D. Matthew. 1918. Contributions to the Snake Creek Fauna with notes upon the Pleistocene of western Nebraska, American Museum Expedition of 1916. *Bulletin of the American Museum of Natural History* **38(7)**:183-229 |
| *Aphelops* | *megalodus* | 18.5-16.3 | 18.5 | X. Wang, R. H. Tedford, and B. E. Taylor. 1999. Phylogenetic systematics of the Borophaginae (Carnivora: Canidae). *Bulletin of the American Museum of Natural History* **243**:1-392 | 12.5-9.4 | 9.4 | W. W. Korth. 1998. Rodents and lagomorphs (Mammalia) from the Late Clarendonian (Miocene) Ash Hollow Formation, Brown County, Nebraska. *Annals of Carnegie Museum* **67(4)**:299-348 |
| *Neohipparion* | *trampasense* | 12.5-9.4 | 12.5 | X. Wang, R. H. Tedford, and B. E. Taylor. 1999. Phylogenetic systematics of the Borophaginae (Carnivora: Canidae). *Bulletin of the American Museum of Natural History* **243**:1-392 | 9.4-4.7 | 4.7 | S. E. Hirschfeld and S. D. Webb. 1968. Plio-Pleistocene Megalonychid Sloths of North America. *Bulletin of the Florida State Museum* **12(5)** |
| *Neohipparion* | *eurystyle* | 11.6-3.6 | 11.6 | D. B. Wright. 1989. Phylogenetic relationships of Catagonus wagneri: sister taxa from the Tertiary of North America. *Advances in Neotropical Mammalogy* 281-308 | 4.7-1.4 | 1.4 | O. Carranza-Castaneda and W. E. Miller. 1993. Hemphillian and Blancan equids from Hidalgo, Mexico. *Journal of Vertebrate Paleontology* **13(3)**:29A |
| *Neohipparion* | *affine* | 12.5-9.4 | 12.5 | M. F. Skinner and F. W. Johnson. 1984. Tertiary stratigraphy and the Frick Collection of fossil vertebrates from north-central Nebraska. Bulletin of the American Museum of Natural History 178(3):1-368 | 11.63-5.333 | 5.333 | S. G. Lucas and G. E. Alvarado. 1995. El proboscideo Rhynchotherium blicki (Mioceno tardio) del oriente de Guatemala. Title translated: The proboscidean Rhynchotherium blicki (upper Miocene) from eastern Guatemala. *Revista Geologica de America Central* **18**:19-24 |
| *Pseudhipparion* | *hessei* | 12.5-9.4 | 12.5 | D. A. Winkler. 1990. Sedimentary Facies and Biochronology of the Upper Tertiary Ogallala Group, Blanco and Yellow House Canyons, Texas Panhandle. In T. C. Gustavson (ed.), *Geologic Framework and Regional Hydrology: Upper Cenozoic Blackwater Draw and Ogallala Formations, Great Plains* 39-55 | 12.5-9.4 | 9.4 | D. A. Winkler. 1990. Sedimentary Facies and Biochronology of the Upper Tertiary Ogallala Group, Blanco and Yellow House Canyons, Texas Panhandle. In T. C. Gustavson (ed.), *Geologic Framework and Regional Hydrology: Upper Cenozoic Blackwater Draw and Ogallala Formations, Great Plains* 39-55 |
| *Pseudhipparion* | *skinneri* | 12.5-9.4 | 12.5 | X. Wang, R. H. Tedford, and B. E. Taylor. 1999. Phylogenetic systematics of the Borophaginae (Carnivora: Canidae). *Bulletin of the American Museum of Natural History* **243**:1-392 | 9.4-4.7 | 4.7 | B. J. MacFadden. 1982. New species of primitive three-toed browsing horse from the Miocene Phosphate Mining District of Central Florida. *Florida Scientist* **45(2)**:117-125 |
| *Merychippus* | *republicanus* | 16.3-12.5 | 16.3 | M. R. Voorhies. 1990. Vertebrate biostratigraphy of the Ogallala Group in Nebraska. In T. C. Gustavson (ed.), *Geologic Framework and Regional Hydrology: Upper Cenozoic Blackwater Draw and Ogallala Formations, Great Plains* 115-151 | 16.3-12.5 | 12.5 | M. R. Voorhies. 1990. Vertebrate biostratigraphy of the Ogallala Group in Nebraska. In T. C. Gustavson (ed.), *Geologic Framework and Regional Hydrology: Upper Cenozoic Blackwater Draw and Ogallala Formations, Great Plains* 115-151 |
| *Cormohipparion* | *johnsoni* | 16.3-9.4 | 16.3 | S. D. Webb. 1969. The Burge and Minnechaduza Clarendonian Mammalian Faunas of North-Central Nebraska. *University of California Publications in Geological Sciences* **78**:1-191 | 16.3-9.4 | 9.4 | S. D. Webb. 1969. The Burge and Minnechaduza Clarendonian Mammalian Faunas of North-Central Nebraska. *University of California Publications in Geological Sciences* **78**:1-191 |
| *Cormohipparion* | *goorisi* | 16.3-12.5 | 16.3 | J. A. Schiebout. 1997. Paleofaunal survey, collecting, processing, and documentation at two locations on Fort Polk, Louisiana. *U.S. Army Corps of Engineers, Fort Worth District; Contract No. DACW63-90-D-0008, Delivery Order 13 [No sponsoring agency report number given]* 1-92 | 16.3-12.5 | 12.5 | J. A. Schiebout. 1997. Paleofaunal survey, collecting, processing, and documentation at two locations on Fort Polk, Louisiana. *U.S. Army Corps of Engineers, Fort Worth District; Contract No. DACW63-90-D-0008, Delivery Order 13 [No sponsoring agency report number given]* 1-92 |
| *Hipparion* | *shirleyae* | 16.3-12.5 | 16.3 | B. J. MacFadden. 1984. Systematics and phylogeny of Hipparion, Neohipparion, Nannippus, and Cormohipparion (Mammalia, Equidae) from the Miocene and Pliocene of the New World. *Bulletin of the American Museum of Natural History* **179(1)**:1-195 | 16.3-12.5 | 12.5 | B. J. MacFadden. 1984. Systematics and phylogeny of Hipparion, Neohipparion, Nannippus, and Cormohipparion (Mammalia, Equidae) from the Miocene and Pliocene of the New World. *Bulletin of the American Museum of Natural History* **179(1)**:1-195 |
| *Protohippus* | *supremus* | 16.3-9.4 | 16.3 | X. Wang, R. H. Tedford, and B. E. Taylor. 1999. Phylogenetic systematics of the Borophaginae (Carnivora: Canidae). *Bulletin of the American Museum of Natural History* **243**:1-392 | 11.6-5.3 | 5.3 | C. S. Johnston and W. G. Christian. 1941. Pliocyon walkerae, a new Pliocene canid from Texas. *Journal of Paleontology* **15(1)**:56-60 |
| *Protohippus* | *vetus* | 16.3-12.5 | 16.3 | C. J. Hesse. 1943. A Preliminary Report on the Miocene Vertebrate Faunas of Southeast Texas. *Transactions of the Texas Academy of Sciences* **26**:157-179 | 16.3-12.5 | 12.5 | C. J. Hesse. 1943. A Preliminary Report on the Miocene Vertebrate Faunas of Southeast Texas. *Transactions of the Texas Academy of Sciences* **26**:157-179 |
| *Calippus* | *regulus* | 16.3-12.5 | 16.3 | C. J. Hesse. 1943. A Preliminary Report on the Miocene Vertebrate Faunas of Southeast Texas. Transactions of the Texas Academy of Sciences 26:157-179 | 12.5-9.4 | 9.4 | R. J. Zakrzewski. 1988. Preliminary report on fossil mammals from the Ogallala (Miocene) of north-central Kansas. Fort Hays Studies, Science, third series 10:117-127 |
| *Calippus* | *martini* | 15.98-13.82 | 15.98 | G. S. Morgan. 1989. Miocene vertebrate faunas from the Suwannee River basin of north Florida and south Georgia. *Southeastern Geological Society Guidebook* **30**:26-53 | 11.6-3.6 | 3.6 | X. Wang, R. H. Tedford, and B. E. Taylor. 1999. Phylogenetic systematics of the Borophaginae (Carnivora: Canidae). *Bulletin of the American Museum of Natural History* **243**:1-392 |
| *Calippus* | *proplacidus* | 16.3-12.5 | 16.3 | M. F. Skinner and F. W. Johnson. 1984. Tertiary stratigraphy and the Frick Collection of fossil vertebrates from north-central Nebraska. *Bulletin of the American Museum of Natural History* **178(3)**:1-368 | 11.63-7.246 | 7.246 | G. S. Morgan and A. E. Pratt. 1988. *Southeastern Geological Society Guidebook* **29** |
| *Acritohippus* | *isonesus* | 18.5-16.3 | 18.5 | R. H. Tedford. 1981. Mammalian biochronology of the late Cenozoic basins of New Mexico. *Geological Society America Bulletin* **92** | 9.4-4.7 | 4.7 | W. D. Kuenzi and R. W. Fields. 1971. Tertiary Stratigraphy, Structure, and Geologic History, Jefferson Basin, Montana. *Geological Society America Bulletin* **82(12)**:3373-3394 |
| *Pliohippus* | *pernix* | 15.98-13.82 | 15.98 | G. S. Morgan. 1989. Miocene vertebrate faunas from the Suwannee River basin of north Florida and south Georgia. Southeastern Geological Society Guidebook 30:26-53 | 12.5-2.58 | 2.58 | N. J. Czaplewski. 2008. Miocene Vertebrates From Ogallala Formation Sites in Western Oklahoma. In S. G. Lucas (ed.), Neogene Mammals (New Mexico Museum of Natural History and Science Bulletin) 44:1-14 |
| *Pliohippus* | *mirabilis* | 16.3-12.5 | 16.3 | M. F. Skinner and F. W. Johnson. 1984. Tertiary stratigraphy and the Frick Collection of fossil vertebrates from north-central Nebraska. Bulletin of the American Museum of Natural History 178(3):1-368 | 11.63-7.246 | 7.246 | G. S. Morgan and A. E. Pratt. 1988. *Southeastern Geological Society Guidebook* **29** |
| *Equus* | *alaskae* | 1.4-0.21 | 1.4 | J. J. Stephens. 1960. Stratigraphy and Paleontology of a late Pleistocene basin, Harper County, Oklahoma. *Geological Society America Bulletin* **71** | 1.4-0.21 | 0.21 | H. A. Semken, Jr. 1966. Stratigraphy and Paleontology of the McPherson Equus Beds (Sandahl Local Fauna), McPherson County, Kansas. *Contributions from the Museum of Paleontology, University of Michigan* **20(6)**:121-178 |
| *Equus* | *simplicidens* | 4.7-1.4 | 4.7 | R. E. Reynolds and E. H. Lindsay. 1999. Late Tertiary basins and vertebrate faunas along the Nevada-Utah border. Utah Geological Survey Miscellaneous Publication 99-1:469-478 | 1.4-0.0117 | 0.0117 | R. E. Reynolds and E. H. Lindsay. 1999. Late Tertiary basins and vertebrate faunas along the Nevada-Utah border. Utah Geological Survey Miscellaneous Publication 99-1:469-478 |
| *Onohippidium* | *galushai* | 9.4-4.7 | 9.4 | B. J. MacFadden, N. M. Johnson, and N. D. Opdyke. 1979. Magnetic polarity stratigraphy of the Mio-Pliocene mammal-bearing Big Sandy Formation of western Arizona. *Earth and Planetary Science Letters* **44(3)**:349-364 | 9.4-4.7 | 4.7 | B. J. MacFadden, N. M. Johnson, and N. D. Opdyke. 1979. Magnetic polarity stratigraphy of the Mio-Pliocene mammal-bearing Big Sandy Formation of western Arizona. *Earth and Planetary Science Letters* **44(3)**:349-364 |
| *Scaphohippus* | *sumani* | 18.5-12.5 | 18.5 | B. E. Stoneburg, A. T. McDonald, A. C. Dooley, Jr., E. Scott, and C. J. Hohman. 2021. New remains of middle Miocene equids from the Cajon Valley Formation, San Bernardino National Forest, San Bernardino County, California, USA. PaleoBios 38:1-10 | 16.3-12.5 | 12.5 | M. F. Skinner, S. M. Skinner, and R. J. Gooris. 1977. Stratigraphy and biostratigraphy of late Cenozoic deposits in central Sioux County, western Nebraska. Bulletin of the American Museum of Natural History 158(5):263-370 |
| *Merychippus* | *primus* | 18.5-16.3 | 18.5 | J. Munthe. 1988. Miocene mammals of the Split Rock area, Granite Mountains basin, central Wyoming. University of California Publications in Geological Sciences 126:1-136 | 16.3-12.5 | 12.5 | J. D. Bryant. 1991. New early Barstovian (middle Miocene) vertebrates from the upper Torreya Formation, eastern Florida panhandle. Journal of Vertebrate Paleontology 11(4):472-489 |
| *Merychippus* | *insignis* | 18.5-16.3 | 18.5 | M. Cassiliano. 1980. Stratigraphy and vertebrate paleontology of the Horse Creek-Trail Creek area, Laramie County, Wyoming. Contributions to Geology, University of Wyoming 19(1):25-58 | 12.5-9.4 | 9.4 | M. F. Skinner, S. M. Skinner, and R. J. Gooris. 1977. Stratigraphy and biostratigraphy of late Cenozoic deposits in central Sioux County, western Nebraska. Bulletin of the American Museum of Natural History 158(5):263-370 |
| *Parahippus* | *cognatus* | 18.5-16.3 | 18.5 | : T. Galusha. 1975. Stratigraphy of the Box Butte Formation, Nebraska. Bulletin of the American Museum of Natural History 156(1) | 16.3-12.5 | 12.5 | M. R. Voorhies. 1990. Vertebrate paleontology of the proposed Norden Reservoir Area, Brown, Cherry and Keya Paha counties, Nebraska. Technical Report, Division of Archeological Research, Department of Anthropology, University of Nebraska 82-09 |
| *Desmatippus* | *integer* | 18.5 - 16.3 | 18.5 | X. Wang, R. H. Tedford, and B. E. Taylor. 1999. Phylogenetic systematics of the Borophaginae (Carnivora: Canidae). Bulletin of the American Museum of Natural History 243:1-392 | 16.3-12.5 | 12.5 | B. E. Taylor and S. D. Webb. 1976. Miocene Leptomerycidae (Artiodactyla, Ruminantia) and their relationships. American Museum Novitates 2596 |
| *Megahippus* | *mckennai* | 16.3 - 12.5 | 16.3 | P. C. Henshaw. 1942. A Tertiary mammalian fauna from the San Antonio Mountains near Tonopah, Nevada. Carnegie Institution of Washington Publication 530(5):77-168 | 16.3 - 12.5 | 12.5 | R. H. Tedford and R. M. Alf. 1962. A New Megahippus From the Barstow Formation San Bernardino County, California. Bulletin of the Southern California Academy of Sciences 61(2):113-122 |
| *Hypohippus* | *equinus* | 18.5-16.3 | 18.5 | T. Skwara. 1988. Mammals of the Topham Local Fauna: Early Miocene (Hemingfordian), Cypress Hills Formation, Saskatechewan. Natural History Contributions 9:1-169 | 18.5-16.3 | 16.3 | T. Skwara. 1988. Mammals of the Topham Local Fauna: Early Miocene (Hemingfordian), Cypress Hills Formation, Saskatechewan. Natural History Contributions 9:1-169 |

Table S1.4 Tip-updating for species included in the lower premolar molar boundary artiodactyls data set as per first and last occurrence data from the PBDB (<https://paleobiodb.org/#/>).

| *Genus* | *Species* | First appearance (MYA) | FAD | Reference | Last appearance (MYA) | LAD | Reference |
| --- | --- | --- | --- | --- | --- | --- | --- |
| *Tapirus* | *johnsoni* | 12.5-9.4 | 12.5 | M. A. Turner. 1972. A faunal assemblage from the Lower Ash Hollow Formation (Neogene) of southern Nebraska. *M. S. thesis University of Nebraska* 1-88 | 12.5-9.4 | 9.4 | M. A. Turner. 1972. A faunal assemblage from the Lower Ash Hollow Formation (Neogene) of southern Nebraska. *M. S. thesis University of Nebraska* 1-88 |
| *Teleoceras* | *proterum* | 11.63 - 7.246 | 11.63 | G. S. Morgan. 1986. The so-called giant Miocene dolphin Megalodelphis magnidens Kellogg (Mammalia: Cetacea) is actually a crocodile (Reptilia: Crocodilia). *Journal of Paleontology* **60(2)**:411-417 | 9.4-4.7 | 4.7 | G. S. Morgan. 1986. The so-called giant Miocene dolphin Megalodelphis magnidens Kellogg (Mammalia: Cetacea) is actually a crocodile (Reptilia: Crocodilia). *Journal of Paleontology* **60(2)**:411-417 |
| *Peraceras* | *profectum* | 18.5-16.3 | 18.5 | M. F. Skinner, S. M. Skinner, and R. J. Gooris. 1977. Stratigraphy and biostratigraphy of late Cenozoic deposits in central Sioux County, western Nebraska. *Bulletin of the American Museum of Natural History* **158(5)**:263-370 | 16.3-9.4 | 9.4 | S. G. Lucas and R. M. Schoch. 1982. Duchesneodus, a New Name for Some Titanotheres (Perissodactyla, Brontotheriidae) from the Late Eocene of Western North America. *Journal of Paleontology* **56(4)**:1018-1023 |
| *Neohipparion* | *trampasense* | 12.5-9.4 | 12.5 | X. Wang, R. H. Tedford, and B. E. Taylor. 1999. Phylogenetic systematics of the Borophaginae (Carnivora: Canidae). *Bulletin of the American Museum of Natural History* **243**:1-392 | 9.4-4.7 | 4.7 | S. E. Hirschfeld and S. D. Webb. 1968. Plio-Pleistocene Megalonychid Sloths of North America. *Bulletin of the Florida State Museum* **12(5)** |
| *Neohipparion* | *eurystyle* | 11.6-3.6 | 11.6 | D. B. Wright. 1989. Phylogenetic relationships of Catagonus wagneri: sister taxa from the Tertiary of North America. *Advances in Neotropical Mammalogy* 281-308 | 4.7-1.4 | 1.4 | O. Carranza-Castaneda and W. E. Miller. 1993. Hemphillian and Blancan equids from Hidalgo, Mexico. *Journal of Vertebrate Paleontology* **13(3)**:29A |
| *Pseudhipparion* | *hessei* | 12.5-9.4 | 12.5 | D. A. Winkler. 1990. Sedimentary Facies and Biochronology of the Upper Tertiary Ogallala Group, Blanco and Yellow House Canyons, Texas Panhandle. In T. C. Gustavson (ed.), *Geologic Framework and Regional Hydrology: Upper Cenozoic Blackwater Draw and Ogallala Formations, Great Plains* 39-55 | 12.5-9.4 | 9.4 | D. A. Winkler. 1990. Sedimentary Facies and Biochronology of the Upper Tertiary Ogallala Group, Blanco and Yellow House Canyons, Texas Panhandle. In T. C. Gustavson (ed.), *Geologic Framework and Regional Hydrology: Upper Cenozoic Blackwater Draw and Ogallala Formations, Great Plains* 39-55 |
| *Pseudhipparion* | *skinneri* | 12.5-9.4 | 12.5 | X. Wang, R. H. Tedford, and B. E. Taylor. 1999. Phylogenetic systematics of the Borophaginae (Carnivora: Canidae). *Bulletin of the American Museum of Natural History* **243**:1-392 | 9.4-4.7 | 4.7 | B. J. MacFadden. 1982. New species of primitive three-toed browsing horse from the Miocene Phosphate Mining District of Central Florida. *Florida Scientist* **45(2)**:117-125 |
| *Nannippus* | *peninsulatus* | 9.4-4.7 | 9.4 | O. Carranza-Castaneda, W. E. Miller, and B. J. Kowallis. 1998. New vertebrate faunas from the Transmexican Volcanic Belt, central Mexico. *Journal of Vertebrate Paleontology* **18(3)**:31A | 2.58-0.0117 | 0.0117 | H. Howard. 1969. Avian fossils from three Pleistocene sites in central Mexico. *Los Angeles County Museum Contributions in Science* **172**:1-11 |
| *Cormohipparion* | *goorisi* | 16.3-12.5 | 16.3 | J. A. Schiebout. 1997. Paleofaunal survey, collecting, processing, and documentation at two locations on Fort Polk, Louisiana. *U.S. Army Corps of Engineers, Fort Worth District; Contract No. DACW63-90-D-0008, Delivery Order 13 [No sponsoring agency report number given]* 1-92 | 16.3-12.5 | 12.5 | J. A. Schiebout. 1997. Paleofaunal survey, collecting, processing, and documentation at two locations on Fort Polk, Louisiana. *U.S. Army Corps of Engineers, Fort Worth District; Contract No. DACW63-90-D-0008, Delivery Order 13 [No sponsoring agency report number given]* 1-92 |
| *Protohippus* | *gidleyi* | 12.5-9.4 | 12.5 | X. Wang, R. H. Tedford, and B. E. Taylor. 1999. Phylogenetic systematics of the Borophaginae (Carnivora: Canidae). *Bulletin of the American Museum of Natural History* **243**:1-392 | 9.4-4.7 | 4.7 | B. J. MacFadden. 1982. New species of primitive three-toed browsing horse from the Miocene Phosphate Mining District of Central Florida. *Florida Scientist* **45(2)**:117-125 |
| *Protohippus* | *supremus* | 16.3-9.4 | 16.3 | X. Wang, R. H. Tedford, and B. E. Taylor. 1999. Phylogenetic systematics of the Borophaginae (Carnivora: Canidae). *Bulletin of the American Museum of Natural History* **243**:1-392 | 11.6-5.3 | 5.3 | C. S. Johnston and W. G. Christian. 1941. Pliocyon walkerae, a new Pliocene canid from Texas. *Journal of Paleontology* **15(1)**:56-60 |
| *Calippus* | *regulus* | 16.3-12.5 | 16.3 | C. J. Hesse. 1943. A Preliminary Report on the Miocene Vertebrate Faunas of Southeast Texas. Transactions of the Texas Academy of Sciences 26:157-179 | 12.5-9.4 | 9.4 | R. J. Zakrzewski. 1988. Preliminary report on fossil mammals from the Ogallala (Miocene) of north-central Kansas. Fort Hays Studies, Science, third series 10:117-127 |
| *Calippus* | *placidus* | 16.3-12.5 | 16.3 | M. R. Voorhies. 1971. Paleoclimatic significance of crocodilian remains from the Ogallala Group (upper Tertiary) in northeastern Nebraska. Journal of Paleontology 45(1):119-121 | 12.5-9.4 | 9.4 | G. E. Schultz. 1990. Stop 14: The Clarendonian faunas of the Texas and Oklahoma panhandles. In T. C. Gustavson (ed.), *Tertiary and Quaternary stratigraphy and vertebrate paleontology of parts of northwestern Texas and eastern New Mexico; Guidebook - Bureau of Economic Geology, University of Texas at Austin* 83-93 |
| *Pliohippus* | *pernix* | 15.98-13.82 | 15.98 | G. S. Morgan. 1989. Miocene vertebrate faunas from the Suwannee River basin of north Florida and south Georgia. Southeastern Geological Society Guidebook 30:26-53 | 12.5-2.58 | 2.58 | N. J. Czaplewski. 2008. Miocene Vertebrates From Ogallala Formation Sites in Western Oklahoma. In S. G. Lucas (ed.), Neogene Mammals (New Mexico Museum of Natural History and Science Bulletin) 44:1-14 |
| *Pliohippus* | *mirabilis* | 16.3-12.5 | 16.3 | M. F. Skinner and F. W. Johnson. 1984. Tertiary stratigraphy and the Frick Collection of fossil vertebrates from north-central Nebraska. Bulletin of the American Museum of Natural History 178(3):1-368 | 11.63-7.246 | 7.246 | G. S. Morgan and A. E. Pratt. 1988. *Southeastern Geological Society Guidebook* **29** |
| *Dinohippus* | *leidyanus* | 9.4-4.7 | 9.4 | B. J. MacFadden, N. M. Johnson, and N. D. Opdyke. 1979. Magnetic polarity stratigraphy of the Mio-Pliocene mammal-bearing Big Sandy Formation of western Arizona. *Earth and Planetary Science Letters* **44(3)**:349-364 | 9.4-4.7 | 4.7 | B. J. MacFadden, N. M. Johnson, and N. D. Opdyke. 1979. Magnetic polarity stratigraphy of the Mio-Pliocene mammal-bearing Big Sandy Formation of western Arizona. *Earth and Planetary Science Letters* **44(3)**:349-364 |
| *Equus* | *simplicidens* | 4.7-1.4 | 4.7 | R. E. Reynolds and E. H. Lindsay. 1999. Late Tertiary basins and vertebrate faunas along the Nevada-Utah border. Utah Geological Survey Miscellaneous Publication 99-1:469-478 | 1.4-0.0117 | 0.0117 | R. E. Reynolds and E. H. Lindsay. 1999. Late Tertiary basins and vertebrate faunas along the Nevada-Utah border. Utah Geological Survey Miscellaneous Publication 99-1:469-478 |
| *Equus* | *scotti* | 0.129-0.0117 | 0.129 | B. H. Slaughter. 1966. The Moore Pit local fauna; Pleistocene of Texas. *Journal of Paleontology* **40(1)**:78-91 | 0.129-0.0117 | 0.0117 | B. H. Slaughter. 1966. The Moore Pit local fauna; Pleistocene of Texas. *Journal of Paleontology* **40(1)**:78-91 |
| *Scaphohippus* | *sumani* | 18.5-12.5 | 18.5 | B. E. Stoneburg, A. T. McDonald, A. C. Dooley, Jr., E. Scott, and C. J. Hohman. 2021. New remains of middle Miocene equids from the Cajon Valley Formation, San Bernardino National Forest, San Bernardino County, California, USA. PaleoBios 38:1-10 | 16.3-12.5 | 12.5 | M. F. Skinner, S. M. Skinner, and R. J. Gooris. 1977. Stratigraphy and biostratigraphy of late Cenozoic deposits in central Sioux County, western Nebraska. Bulletin of the American Museum of Natural History 158(5):263-370 |
| *Merychippus* | *calamarius* | 16.3-12.5 | 16.3 | P. C. Henshaw. 1942. A Tertiary mammalian fauna from the San Antonio Mountains near Tonopah, Nevada. Carnegie Institution of Washington Publication 530(5):77-168 | 12.5-9.4 | 9.4 | M. A. Turner. 1972. A faunal assemblage from the Lower Ash Hollow Formation (Neogene) of southern Nebraska. M. S. thesis University of Nebraska 1-88 |
| *Desmatippus* | *integer* | 18.5 - 16.3 | 18.5 | X. Wang, R. H. Tedford, and B. E. Taylor. 1999. Phylogenetic systematics of the Borophaginae (Carnivora: Canidae). Bulletin of the American Museum of Natural History 243:1-392 | 16.3-12.5 | 12.5 | B. E. Taylor and S. D. Webb. 1976. Miocene Leptomerycidae (Artiodactyla, Ruminantia) and their relationships. American Museum Novitates 2596 |
| *Desmatippus* | *crenidens* | 16.3-12.5 | 16.3 | Tedford, R. H., Albright, L. B., Barnosky, A. D., Ferrusquia-Villafranca, I., Hunt, R. M., Storer, J. E., ... & Whistler, D. P. (2004). Mammalian biochronology of the Arikareean through Hemphillian interval (late Oligocene through early Pliocene epochs). In *Late Cretaceous and Cenozoic mammals of North America: biostratigraphy and geochronology* (pp. 169-231). Columbia University Press. | 16.3-12.5 | 12.5 | Tedford, R. H., Albright, L. B., Barnosky, A. D., Ferrusquia-Villafranca, I., Hunt, R. M., Storer, J. E., ... & Whistler, D. P. (2004). Mammalian biochronology of the Arikareean through Hemphillian interval (late Oligocene through early Pliocene epochs). In *Late Cretaceous and Cenozoic mammals of North America: biostratigraphy and geochronology* (pp. 169-231). Columbia University Press. |
| *Archaeohippus* | *mourningi* | 18.5-16.3 | 18.5 | R. E. Reynolds. 1991. Biostratigraphic relationships of Tertiary small vertebrates from Cajon Valley, San Bernardino County, California. In M.O. Woodburne, R.E. Reynolds, and D.P. Whistler, (eds.), San Bernardino County Museum Association Quarterly 38(3):54-59 | 15.98-11.63 | 11.63 | C. A. Repenning and J. G. Vedder. 1961. Continental vertebrates and their stratigraphic correlation with marine mollusks, eastern Caliente Range, California. United States Geological Survey Professional Paper 424C(235):C-235-C239 |
| *Megahippus* | *mckennai* | 16.3 - 12.5 | 16.3 | P. C. Henshaw. 1942. A Tertiary mammalian fauna from the San Antonio Mountains near Tonopah, Nevada. Carnegie Institution of Washington Publication 530(5):77-168 | 16.3 - 12.5 | 12.5 | R. H. Tedford and R. M. Alf. 1962. A New Megahippus From the Barstow Formation San Bernardino County, California. Bulletin of the Southern California Academy of Sciences 61(2):113-122 |
| *Megahippus* | *matthewi* | 16.3 - 12.5 | 16.3 | M. F. Skinner and F. W. Johnson. 1984. Tertiary stratigraphy and the Frick Collection of fossil vertebrates from north-central Nebraska. Bulletin of the American Museum of Natural History 178(3):1-368 | 12.5-9.4 | 9.4 | R. H. Tedford and S. F. Barghoorn. 1993. Road Log Neogene geology of the Española Basin, New Mexico. New Mexico Museum of Natural History Bulletin 2:169-178 |
| *Hypohippus* | *equinus* | 18.5-16.3 | 18.5 | T. Skwara. 1988. Mammals of the Topham Local Fauna: Early Miocene (Hemingfordian), Cypress Hills Formation, Saskatechewan. Natural History Contributions 9:1-169 | 18.5-16.3 | 16.3 | T. Skwara. 1988. Mammals of the Topham Local Fauna: Early Miocene (Hemingfordian), Cypress Hills Formation, Saskatechewan. Natural History Contributions 9:1-169 |
| *Parahippus* | *cognatus* | 18.5-16.3 | 18.5 | : T. Galusha. 1975. Stratigraphy of the Box Butte Formation, Nebraska. Bulletin of the American Museum of Natural History 156(1) | 16.3-12.5 | 12.5 | M. R. Voorhies. 1990. Vertebrate paleontology of the proposed Norden Reservoir Area, Brown, Cherry and Keya Paha counties, Nebraska. Technical Report, Division of Archeological Research, Department of Anthropology, University of Nebraska 82-09 |
| *Hypohippus* | *osborni* | 18.5-16.3 | 18.5 | E. C. Galbreath. 1953. A contribution to the Tertiary geology and paleontology of northeastern Colorado. University of Kansas Paleontological Contributions Vertebrata 4:1-120 | 16.3-12.5 | 12.5 | J. F. Sutton and W. W. Korth. 1995. Rodents (Mammalia) from the Barstovian (Miocene) Anceney local fauna, Montana. Annals of Carnegie Museum 64(4):267-314 |
| *Protohippus* | *perditus* | 16.3-12.5 | 16.3 | C. J. Hesse. 1943. A Preliminary Report on the Miocene Vertebrate Faunas of Southeast Texas. Transactions of the Texas Academy of Sciences 26:157-179 | 9.4-4.7 | 4.7 | G. E. Schultz. 1990. Stop 16: Late Hemphillian faunas of the Texas and Oklahoma panhandles. In T. C. Gustavson (ed.), Tertiary and Quaternary stratigraphy and vertebrate paleontology of parts of northwestern Texas and eastern New Mexico; Guidebook - Bureau of Economic Geology, University of Texas at Austin 104-111 |
| *Merychippus* | *insignis* | 18.5-16.3 | 18.5 | M. Cassiliano. 1980. Stratigraphy and vertebrate paleontology of the Horse Creek-Trail Creek area, Laramie County, Wyoming. Contributions to Geology, University of Wyoming 19(1):25-58 | 12.5-9.4 | 9.4 | M. F. Skinner, S. M. Skinner, and R. J. Gooris. 1977. Stratigraphy and biostratigraphy of late Cenozoic deposits in central Sioux County, western Nebraska. Bulletin of the American Museum of Natural History 158(5):263-370 |
| *Aphelops* | *malacorhinus* | 15.98-5.333 | 15.98 | L. B. Albright, A. E. Sanders, R. E. Weems, D. J. Cicimurri, and J. L. Knight. 2019. Cenozoic vertebrate biostratigraphy of South Carolina, U.S.A. and additions to the fauna. Bulletin of the Florida Museum of Natural History 57(2):77-236 | 9.4-4.7 | 4.7 | G. E. Schultz. 1990. Stop 15: Early Hemphillian faunas of the Texas and Oklahoma panhandles. In T. C. Gustavson (ed.), Tertiary and Quaternary stratigraphy and vertebrate paleontology of parts of northwestern Texas and eastern New Mexico; Guidebook - Bureau of Economic Geology, University of Texas at Austin 95-103 |
| *Merychippus* | *primus* | 18.5-16.3 | 18.5 | J. Munthe. 1988. Miocene mammals of the Split Rock area, Granite Mountains basin, central Wyoming. University of California Publications in Geological Sciences 126:1-136 | 16.3-12.5 | 12.5 | J. D. Bryant. 1991. New early Barstovian (middle Miocene) vertebrates from the upper Torreya Formation, eastern Florida panhandle. Journal of Vertebrate Paleontology 11(4):472-489 |
