# Supplementary material for: You Should Look a Gift Ungulate in the Mouth: Using 2D Occlusal Cheek Tooth Morphology to Study the Evolution of Molarization in Ungulates
Source: Integr Org Biol. 2026 May 30;8(1):obag025. doi: 10.1093/iob/obag025 (PMC13266073; doi:10.1093/iob/obag025)
Supplement: obag025_Supplemental_Files [file obag025_supplemental_files.zip › IOB_2026-006_SUPPLEMENTARY FILE_2.docx]

| Genus | Species |
| --- | --- |
| *Antilocapra* | *americana* |
| *Bison* | priscus |
| *Cervalces* | *latifrons* |
| *Merychippus* | *insignis* |
| *Ovis* | aries |
| *Parahippus* | *cognatus* |
| *Procranioceras* | skinneri |
| *Protohippus* | *perditus* |
| *Pseudoceras* | *skinneri* |
| *Ramoceros* | *osborni* |
| *Oreamnos* | *americanus* |
| *Ovis* | *canadensis* |
| *Ovis* | *dalli* |
| *Ovibos* | *moschatus* |
| *Bison* | *bison* |
| *Cervus* | *elaphus* |
| *Odocoileus* | *virginianus* |
| *Odocoileus* | *hemionus* |
| *Rangifer* | *tarandus* |
| *Alces* | *alces* |
| *Saiga* | *tatarica* |
| *Bison* | *antiquus* |
| *Merycerus* | *warreni* |
| *Ramoceros* | *ramosus* |
| *Merycodus* | *sabulonis* |
| *Cosoryx* | *furcatus* |
| *Capromeryx* | *furcifer* |
| *Capromeryx (Texoceros)* | *minorei* |
| *Osbornoceros* | *osborni* |
| *Paracosoryx* | *wilsoni* |
| *Ilingoceros* | *alexandrae* |
| *Paracosoryx* | *alticornis* |
| *Merriamoceros* | *coronatus* |
| *Miolabis* | *tenuis* |
| *Miolabis* | *princetonianus* |
| *Miolabis* | *fissidens* |
| *Aepycamelus* | *major* |
| *Megatylopus* | *gigas* |
| *Palaeolama* | *mirifica* |
| *Cranioceras* | *unicornis* |
| *Platygonus* | *compressus* |
| *Aptenohyus / Prosthennops* | *niobrarensis* |
| *Macrogenis / Prosthennops* | *crassigenis* |
| *Prosthennops* | *serus* |
| *Cynorca* | *occidentale* |
| *Dromomeryx* | *borealis* |
| *Rakomeryx* | *sinclairi* |
| *Longirostromeryx* | *wellsi* |
| *Longirostromeryx* | *clarendonensis* |
| *Blastomeryx* | *gemmifer* |
| *Merychippus* | *calamarius* |
| *Scaphohippus* | *sumani* |
| *Megahippus* | *matthewi* |
| *Hypohippus* | *equinus* |
| *Desmatippus* | *integer* |
| *Megahippus* | *mckennai* |
| *Archaeohippus* | *mourningi* |
| *Desmatippus* | *crenidens* |
| *Neohipparion* | *trampasense* |
| *Neohipparion* | *eurystyle* |
| *Nannippus* | *peninsulatus* |
| *Cormohipparion* | *goorisi* |
| *Pseudhipparion* | *skinneri* |
| *Pseudhipparion* | *hessei* |
| *Pliohippus* | *mirabilis* |
| *Calippus* | *regulus* |
| *Calippus* | *placidus* |
| *Protohippus* | *gidleyi* |
| *Protohippus* | *supremus* |
| *Pliohippus* | *pernix* |
| *Dinohippus* | *leidyanus* |
| *Equus* | *simplicidens* |
| *Equus* | *scotti* |
| *Peraceras* | *profectum* |
| *Tapirus* | *johnsoni* |
| *Teleoceras* | *proterum* |
| *Oreamnos* | *americanus* |
| *Ovis* | *dalli* |
| *Bison* | *bison* |
| *Cervus* | *elaphus* |
| *Odocoileus* | *virginianus* |
| *Odocoileus* | *hemionus* |
| *Rangifer* | *tarandus* |
| *Alces* | *alces* |
| *Merycodus* | *sabulonis* |
| *Cosoryx* | *furcatus* |
| *Cosoryx* | *ilfonsensis* |
| *Paracosoryx* | *wilsoni* |
| *Merriamoceros* | *coronatus* |
| *Merychippus* | *republicanus* |
| *Merychippus* | *insignis* |
| *Scaphohippus* | *sumani* |
| *Merychippus* | *primus* |
| *Parahippus* | *cognatus* |
| *Hypohippus* | *equinus* |
| *Desmatippus* | *integer* |
| *Megahippus* | *mckennai* |
| *Neohipparion* | *affine* |
| *Neohipparion* | *trampasense* |
| *Neohipparion* | *eurystyle* |
| *Cormohipparion* | *goorisi* |
| *Hipparion* | *shirleyae* |
| *Pseudhipparion* | *skinneri* |
| *Pseudhipparion* | *hessei* |
| *Pliohippus* | *mirabilis* |
| *Calippus* | *regulus* |
| *Calippus* | *proplacidus* |
| *Protohippus* | *supremus* |
| *Calippus* | *martini* |
| *Pliohippus* | *pernix* |
| *Hippotherium* | *isonesum* |
| *Cormohipparion* | *johnsoni* |
| *Protohippus* | *vetus* |
| *Equus* | *alaskae* |
| *Onohippidium* | *galushai* |
| *Equus* | *simplicidens* |
| *Miolabis* | *tenuis* |
| *Pleiolama* | *mckennai* |
| *Platygonus* | *compressus* |
| *Aptenohyus / Prosthennops* | *niobrarensis* |
| *Peraceras* | *superciliosum* |
| *Tapirus* | *johnsoni* |
| *Aphelops* | *megalodus* |
| *Ticholeptus* | *zygomaticus* |
|  |  |
|  |  |
|  |  |
|  |  |
|  |  |
