# Supplementary material for: You Should Look a Gift Ungulate in the Mouth: Using 2D Occlusal Cheek Tooth Morphology to Study the Evolution of Molarization in Ungulates
Source: Integr Org Biol. 2026 May 30;8(1):obag025. doi: 10.1093/iob/obag025 (PMC13266073; doi:10.1093/iob/obag025)
Supplement: obag025_Supplemental_Files [file obag025_supplemental_files.zip › IOB_2026-006_SUPPLEMENTARY FILE_3.docx]

Table S3.1 Description of the landmark scheme generated including the number, tooth the landmark was placed on, the corresponding type of landmark sensu Bookstein (1997), and the detailed description. Colour coding correspond to the type of cusp/cuspid the landmarking occurred on; blue = Mesiobuccal, orange = Mesiolingual, yellow = Distobuccal, green = Distolingual.

| **Landmark number** | **Tooth** | **Type** | **Description** |
| --- | --- | --- | --- |
| 1 | p4 | II | Mesiobuccal cusp/cuspid apex |
| 2 | p4 | II | Mesiolingual cusp/cuspid apex |
| 3 | p4 | II | Distobuccal cusp/cuspid apex |
| 4 | p4 | II | Distolingual cusp/cuspid apex |
| 5 | m1 | II | Mesiobuccal cusp/cuspid apex |
| 6 | m1 | II | Mesiolingual cusp/cuspid apex |
| 7 | m1 | II | Distobuccal cusp/cuspid apex |
| 8 | m1 | II | Distolingual cusp/cuspid apex |
| 9 | N/A | I | Most acute buccal angle between the p4 and m1 |
| 10 | N/A | I | Most acute lingual angle between the p4 and m1 |
| 11 | p4 | III | Maximum buccal extension of the mesiobuccal cusp/cuspid parallel to LM 1 |
| 12 to 15 | p4 | Sliding III | Resampled SLM along a curve between LM 11 and 16 |
| 16 | p4 | III | Maximum curvature of mesiobuccal cusp/cuspid anterior to LM 11 |
| 17 | p4 | III | Maximum buccal extension of the mesiolingual cusp/cuspid parallel to LM 2 |
| 18 to 21 | p4 | Sliding III | Resampled SLM along a curve between LM 17 and 22 |
| 22 | p4 | III | Maximum curvature of mesiolingual cusp/cuspid anterior to LM 17 |
| 23 | p4 | III | Maximum buccal extension of the distolingual cusp/cuspid parallel to LM 8 |
| 24 to 27 | p4 | Sliding III | Resampled SLM along a curve between LM 53 and 58 |
| 28 | p4 | III | Maximum curvature of distolingual cusp/cuspid posterior to LM 53 |
| 29 | m1 | III | Maximum buccal extension of the mesiolingual cusp/cuspid parallel to LM 6 |
| 30 to 33 | m1 | Sliding III | Resampled SLM along a curve between LM 41 and 46 |
| 34 | m1 | III | Maximum curvature of mesiolingual cusp/cuspid anterior to LM 41 |
| 35 | m1 | III | Maximum buccal extension of the distolingual cusp/cuspid parallel to LM 4 |
| 36 to 39 | m1 | Sliding III | Resampled SLM along a curve between LM 29 and 34 |
| 40 | m1 | III | Maximum curvature of distolingual cusp/cuspid posterior to LM 29 |
| 41 | m1 | III | Maximum buccal extension of the distobuccal cusp/cuspid parallel to LM 7 |
| 42 to 45 | m1 | Sliding III | Resampled SLM along a curve between LM 47 and 52 |
| 46 | m1 | III | Maximum curvature of mesiolingual cusp/cuspid posterior to LM 47 |
| 47 | m1 | III | Maximum buccal extension of the mesiobuccal cusp/cuspid parallel to LM 5 |
| 48 to 51 | m1 | Sliding III | Resampled SLM along a curve between LM 35 and 40 |
| 52 | m1 | III | Maximum curvature of mesiobuccal cusp/cuspid anterior to LM 35 |
| 53 | p4 | III | Maximum buccal extension of the distobuccal cusp/cuspid parallel to LM 3 |
| 54 to 57 | p4 | Sliding III | Resampled SLM along a curve between LM 23 and 28 |
| 58 | p4 | III | Maximum curvature of mesiolingual cusp/cuspid posterior to LM 23 |


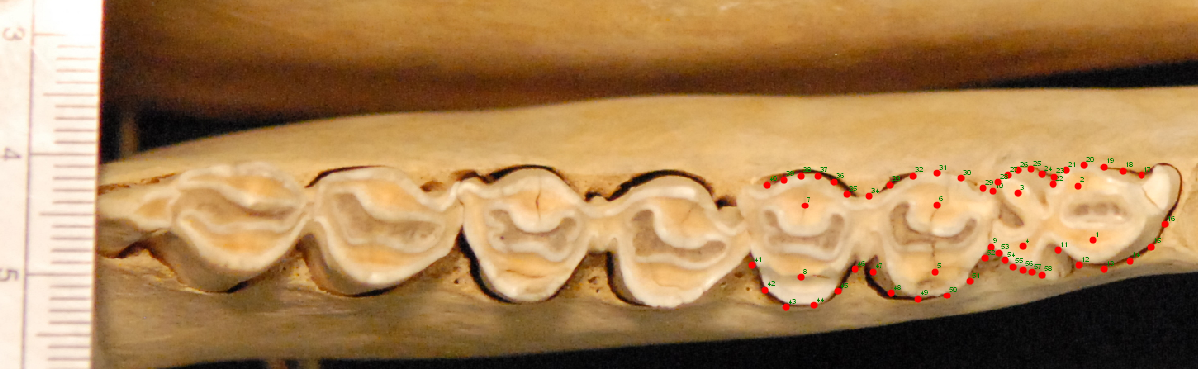


Figure S3.1 – Application of the landmark scheme to generalized ungulate dentition. Mesial is towards the right, lingual is towards the top of the image. Numbers correspond to landmark identity as listed in online Table S3.1.


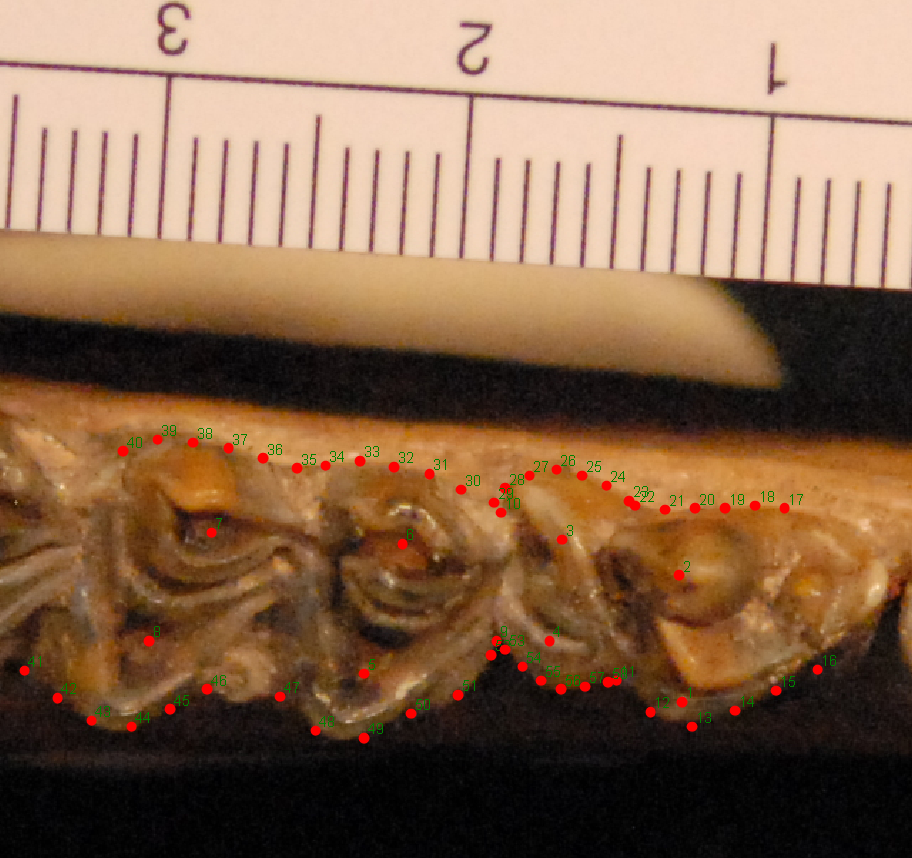


Figure S3.2 – Application of the landmark scheme to selenodont dentition. Mesial is towards the right, lingual is towards the top of the image. Numbers correspond to landmark identity as listed in online Table S3.1.


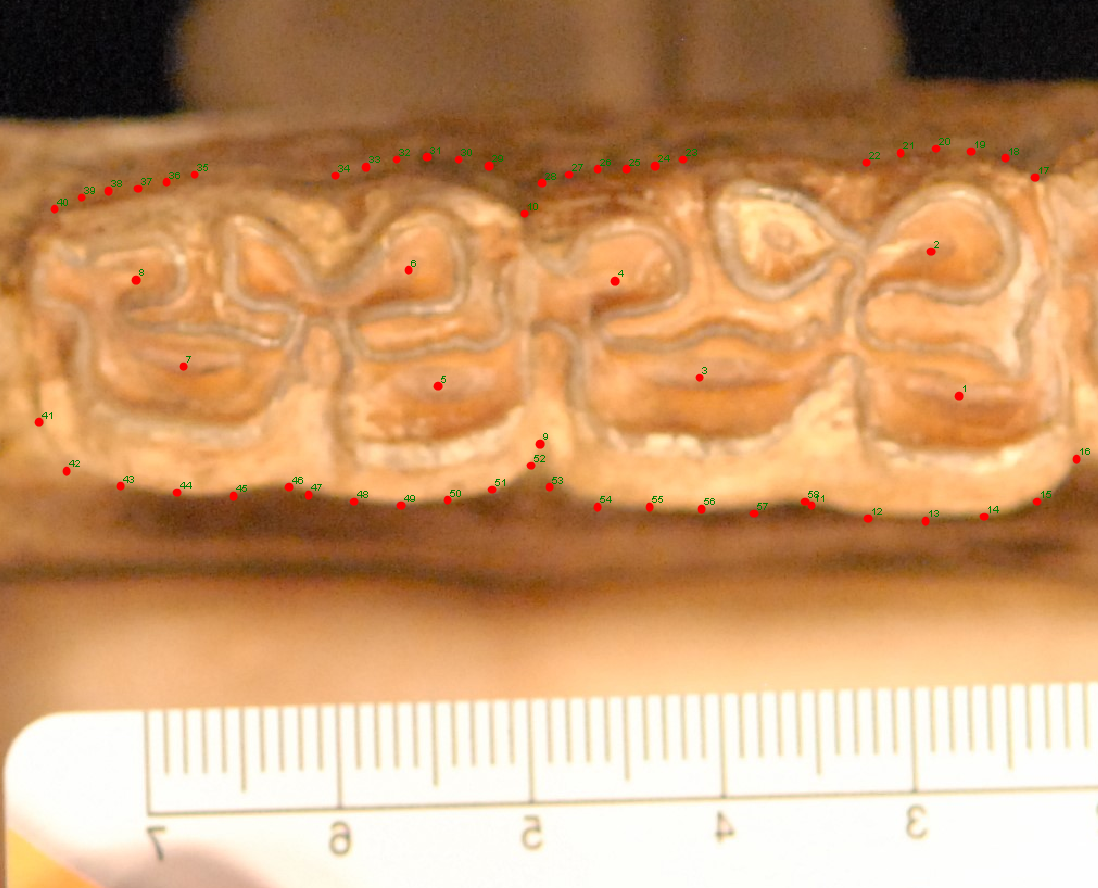


Figure S3.3 – Application of the landmark scheme to equiid dentition. Mesial is towards the right, lingual is towards the top of the image. Numbers correspond to landmark identity as listed in online Table S3.1.


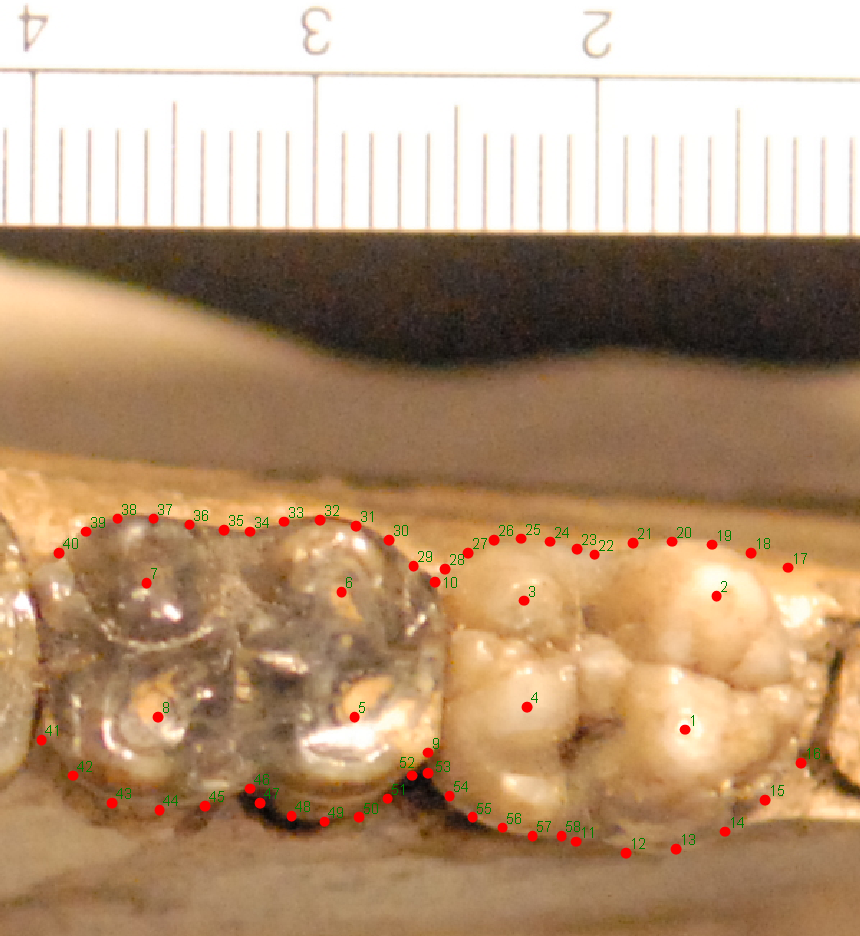


Figure S3.4 – Application of the landmark scheme to bunodont dentition. Mesial is towards the right, lingual is towards the top of the image. Numbers correspond to landmark identity as listed in online Table S3.1.


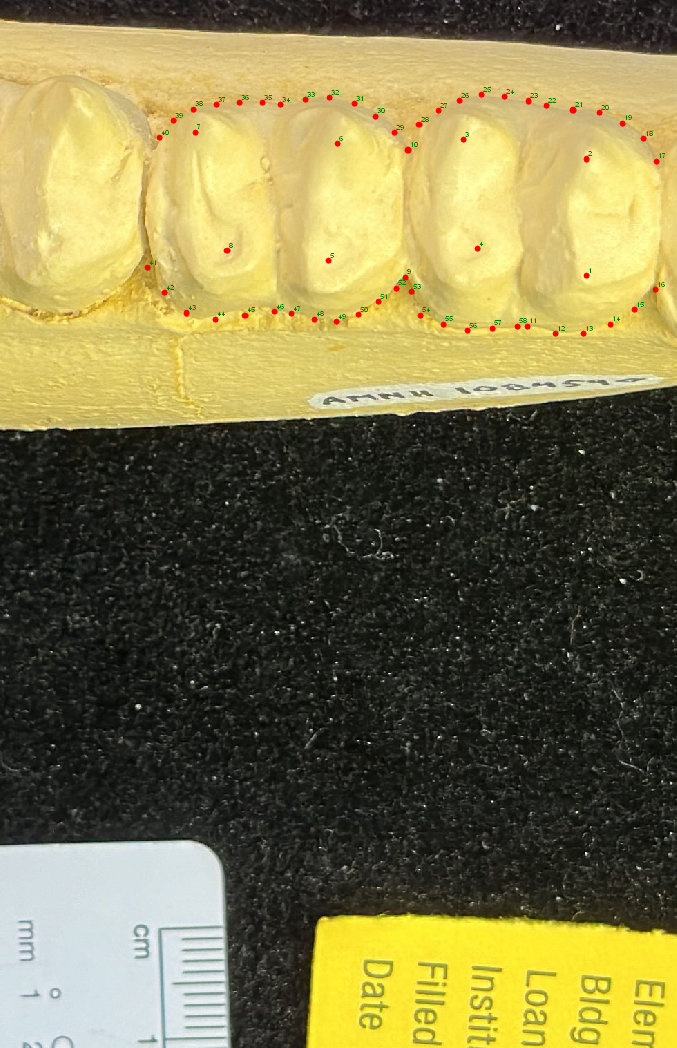


Figure S3.5 – Application of the landmark scheme to lophodont dentition. Mesial is towards the right, lingual is towards the top of the image. Numbers correspond to landmark identity as listed in online Table S3.1.
