# Supplementary material for: You Should Look a Gift Ungulate in the Mouth: Using 2D Occlusal Cheek Tooth Morphology to Study the Evolution of Molarization in Ungulates
Source: Integr Org Biol. 2026 May 30;8(1):obag025. doi: 10.1093/iob/obag025 (PMC13266073; doi:10.1093/iob/obag025)
Supplement: obag025_Supplemental_Files [file obag025_supplemental_files.zip › IOB_2026-006_SUPPLEMENTARY FILE_4.docx]

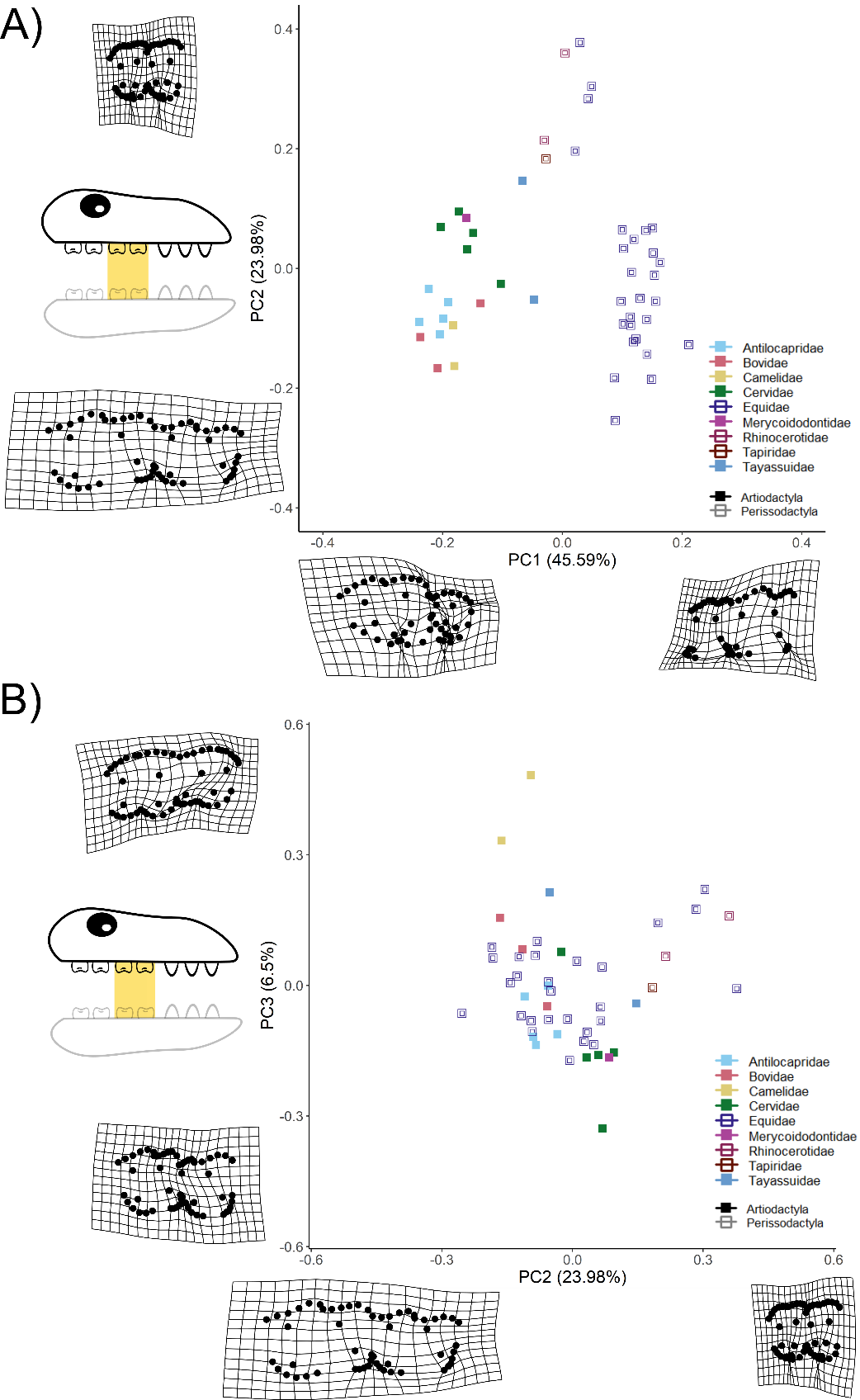


Figure S4.1 - Principal components analysis for differences at the upper premolar molar boundary shape among artiodactyls and perissodactyls (**A**) PC1 versus PC2, (**B**) PC2 versus PC3.


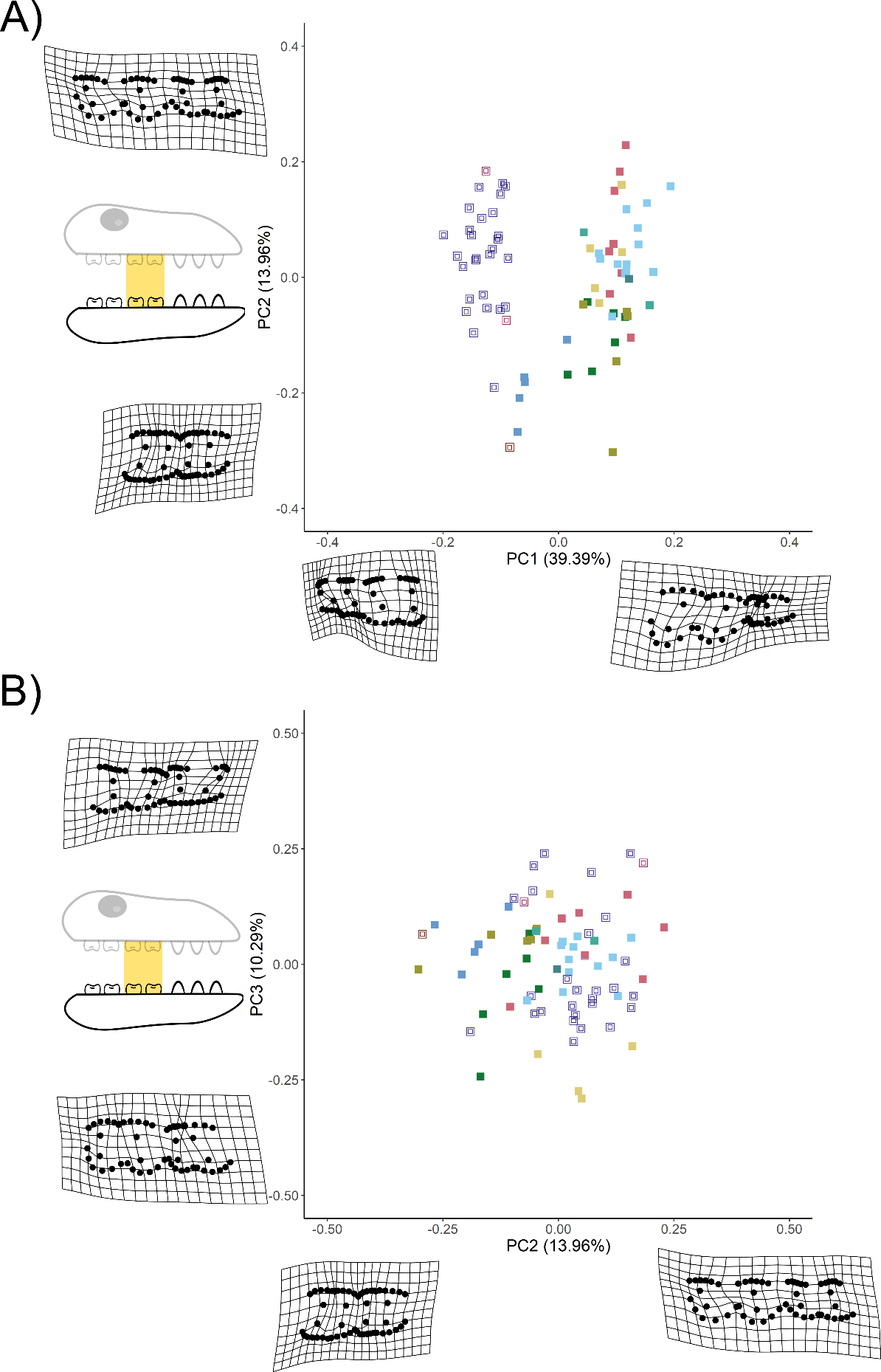


Figure S4.2 - Principal components analysis for differences at the lower premolar molar boundary shape among artiodactyls and perissodactyls (**A**) PC1 versus PC2, (**B**) PC2 versus PC3.
